# Mitochondrial quality control in the brain: The physiological and pathological roles

**DOI:** 10.3389/fnins.2022.1075141

**Published:** 2022-12-12

**Authors:** Xurui Shen, Peixin Sun, Hao Zhang, Hanting Yang

**Affiliations:** Institute for Translational Brain Research, State Key Laboratory of Medical Neurobiology, MOE Frontiers Center for Brain Science, Department of Neurology, Zhongshan Hospital, Fudan University, Shanghai, China

**Keywords:** mitochondrial quality control, mitochondrial dysfunction, brain disorders, mitochondrial homeostasis, therapeutic target

## Abstract

The human brain has high energetic expenses and consumes over 20% of total oxygen metabolism. Abnormal brain energy homeostasis leads to various brain diseases. Among multiple factors that contribute to these diseases, mitochondrial dysfunction is one of the most common causes. Maintenance of mitochondrial integrity and functionality is of pivotal importance to brain energy generation. Mitochondrial quality control (MQC), employing the coordination of multiple mechanisms, is evolved to overcome many mitochondrial defects. Thus, not surprisingly, aberrant mitochondrial quality control results in a wide range of brain disorders. Targeting MQC to preserve and restore mitochondrial function has emerged as a promising therapeutic strategy for the prevention and treatment of brain diseases. Here, we set out to summarize the current understanding of mitochondrial quality control in brain homeostasis. We also evaluate potential pharmaceutically and clinically relevant targets in MQC-associated brain disorders.

## Introduction

The brain is the most important and intricate component of the central nervous system (CNS). In addition to controlling how the body moves, it regulates higher neural activities including spirit, language, learning, memory, and consciousness. Brain damage causes reduced body function, such as memory loss, cognitive impairment, sensory deficits, and behavioral abnormalities. Brain disorders, from neurodegenerative to psychiatric illnesses, have drawn increasing attention in recent years (VanItallie, 2019; Xie et al., 2019; Oh et al., 2021; Wang et al., 2021).

**FIGURE 1:**
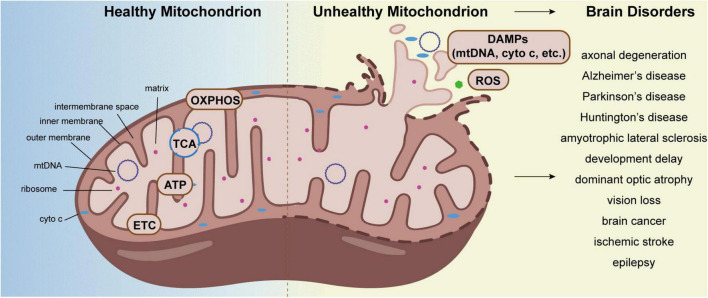
Aberrant mitochondrion causes brain disorders. The tricarboxylic acid cycle (TCA cycle), oxidative phosphorylation (OXPHOS), electron transport chain (ETC), and ATP synthesis all take place primarily in mitochondria. Under stress, mitochondria produce DAMPs and excessive ROS, which cause a variety of brain disorders, including axonal degeneration, neurodegeneration diseases, dominant optic atrophy, epilepsy, and so on.

In humans, the brain accounts for approximately 2% of the body weight, while it consumes over 20% of the body’s energy needs (Ambekar et al., 2021). It is well-known that mitochondria are the center of energy metabolism. They are essential for brain metabolism, development, and function. Although a variety of factors contribute to brain disorders, evidence postulates that mitochondrial dysfunction is one of the leading causes (Guntuku et al., 2016; Lan et al., 2022). Unhealthy and aged brains often show aberrant mitochondrial structures and excessive reactive oxygen species (ROS), which is related to many adult-onset brain diseases, ranging from injuries and infections to brain tumors and dementia (Figure 1; Sultana et al., 2011; Chouchani et al., 2014; Zorov et al., 2014; Cheng et al., 2020; Iranmanesh et al., 2021). Accordingly, the maintenance of mitochondrial homeostasis is crucial for brain function. Cell employed numerous strategies to coordinate protein and organellar quality control, including mechanisms to monitor the mitochondria. In this review, we discuss the pathways of mitochondrial quality control (MQC) and its role in the progression of brain diseases, and briefly summarized the known MQC-related potential drug targets.

## Physiological functions of mitochondria in the brain

### ATP production, metabolism, and oxidative phosphorylation

Mitochondria participate in energy and free radicals production, cell metabolism, cell death, and inflammation in the brain (Martin, 2010; Yin et al., 2016; Stefanatos and Sanz, 2018; Bader and Winklhofer, 2020). Mitochondria are the primary sites of ATP production as well as catabolic biochemical processes such as glycolysis, tricarboxylic acid (TCA) cycle, and oxidative phosphorylation (OXPHOS). At synapses, neurons in the brain exchange chemical and electrical signals with one another. Maintaining electrochemical gradients, liberating and recycling synaptic vesicles, and other very energy-intensive procedures rely on mitochondrial ATP synthesis (Devine and Kittler, 2018). It has been predicted that axonal terminals consume 4.5 × 10^8^ ATP during an action potential (and downstream synaptic events), compared to 3 × 10^6^ ATP used by resting potentials and housekeeping (Harris et al., 2012). The ATP-dependent membrane pumps, such as Na^+^/K^+^ ATPase and Ca^2+^ ATPase, are powered by about 55% of the total ATP produced by neurons in order to maintain the resting potential by resetting ionic gradients (Harris and Attwell, 2012). In addition, synaptic vesicle recycling also consumes a significant amount of energy. Each glutamate synaptic vesicle recycling event requires more than 2 × 10^4^ ATP molecules, and in order to restore ionic gradients at a steady state, 1 × 10^6^ ATP molecules must be restored within each individual neuron terminal (Rangaraju et al., 2014). Besides that, the process of cargo transportation along axons, which is carried out by motors, kinesins, and cytoplasmic dynein, is also ATP-dependent (Gibbs et al., 2015). Therefore, mitochondrial ATP synthesis is essential to keep the brain functioning normally.

Glucose serves as the main source of energy in neurons. Initially, glucose catabolism generates pyruvate, which is then transferred to mitochondria for TCA and OXPHOS (Mergenthaler et al., 2013). By combining electron transport with the phosphorylation of ADP on the inner mitochondrial membrane (IMM), OXPHOS produces ATP. NADH CoQ reductase (complex I), succinate dehydrogenase (complex II), ubiquinol-cytochrome c reductase (complex III), cytochrome c oxidase (complex IV), and ATP synthase (complex V) are all involved in the process (Sousa et al., 2018; Vercellino and Sazanov, 2022).

### Free radicals

Free radicals, particularly ROS, are generated by mitochondria. ROS acts a significant role in the regulation of multiple neuronal cell life processes, including nucleic acid oxidation, immune response, and NF-κB pathway. The major source of free radicals, also known as “mitochondrial ROS,” is the electron transport chain (ETC). There is a strong correlation between the rate of ROS production, mitochondrial membrane potential (MMP), and the activity of the ETC complexes (Islam, 2017; Kalpage et al., 2019).

### Cell death

To maintain organ size and function, mitochondria are required for cell death processes such as apoptosis, necroptosis, pyroptosis, and ferroptosis (Bock and Tait, 2020). The most widely understood mitochondria-related mechanism among them is apoptosis. Mitochondrial apoptosis also referred to as intrinsic apoptosis, is dependent on mitochondrial outer membrane permeabilization (MOMP). The procedure enables the release of proteins from the mitochondrial intermembrane space into the cytoplasm, which causes cell death. The establishment of functional circuitry, upkeep of healthy cell bodies and axons, promotion of myelination, and effective synaptic contact with target muscle are all facilitated by mitochondrial apoptosis in the brain (Buss et al., 2006; Fricker et al., 2018).

### Neuroinflammation

Under certain stress conditions, the outer and inner membrane of mitochondria are damaged, and mitochondrial components such as mitochondrial DNA (mtDNA), formyl peptides, cytochrome c (cyto c) and cardiolipin are released into the cytoplasm, which is regarded as danger-associated molecular patterns (DAMPs), inducing the assembly and activation of the inflammasome, the release of cytokines and the elicitation of innate immune responses (Bader and Winklhofer, 2020). DAMPs released by mitochondria activate microglia in the brain, which represent the primary form of immune defense. In addition to oxidative stress, metabolism, and OXPHOS regulation, mitochondria play an important role in neuroinflammation (Regen et al., 2017; Gu et al., 2021; Zhao et al., 2021).

## Mitochondrial quality control in the brain

**FIGURE 2:**
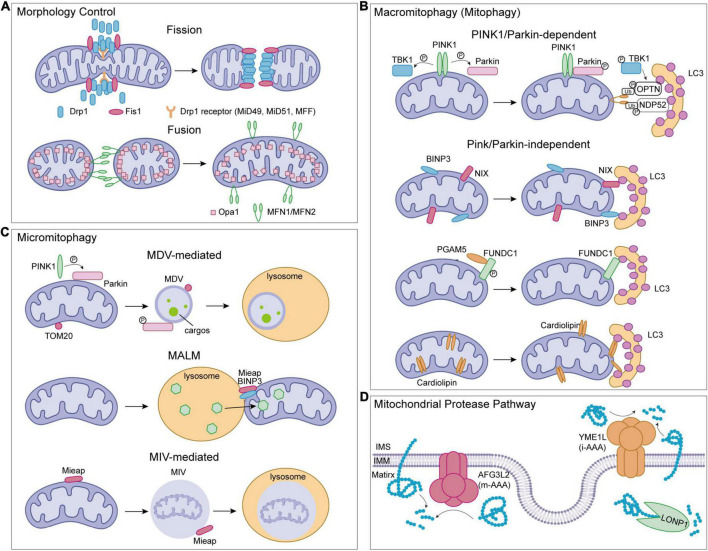
Mitochondrial quality control (MQC) pathways in human. **(A)** Mitochondrial morphology control. **(B)** Mitophagy pathways. **(C)** Micromitophagy pathways. **(D)** Mitochondrial protease pathways. Drp1, dynamic-related protein 1; Fis1, fission 1; MFF, mitochondrial fission factor; MiD49/MiD51, mitochondrial dynamics proteins 49/51; OPA1, optic atrophy 1; MFN1/MFN2, mitofusin1/mitofusin2; PINK1, serine/threonine-protein kinase PINK1; Parkin, E3 ubiquitin-protein ligase parkin; LC3, microtubule-associated protein light chain 3; TBK1, TANK binding kinase 1; NIX, NIP3-like protein X; BNIP3, Bcl-2/adenovirus E1B 19 kDa interacting protein 3; FUNDC1, FUN14 domain containing 1; PGAM5, phosphoglycerate mutase family member 5 phosphatase; Mieap, spermatogenesis-associated protein 18; TOM20, translocase of outer mitochondrial membrane 20; MDV, mitochondrial-derived vesicles; MALM, Mieap-induced accumulation of lysosome-like organelles within mitochondria; MIV, Mieap-induced vacuoles.

Mitochondrial dysfunction causes various diseases in the brain. Mitochondria are semi-autonomous organelles, and their proteome includes about 1,500 human proteins, which are derived from the nuclear genome and mitochondrial genome (Morgenstern et al., 2017). Among them, only 13 proteins are encoded by the mitochondrial genome. About 99% of mitochondrial proteins are synthesized by cytosolic ribosomes, followed by sorted and imported to mitochondria. Mitochondria are the central sites for the development of the TCA and energy production, while also participates in cell metabolism, cell growth, cell death, inflammation, and cell homeostasis. Therefore, MQC mechanisms are essential to ensure proper protein folding and maintain a normal mitochondrial environment. The processes of MQC include mitochondrial morphology control (fission and fusion), macromitophagy (mitophagy), micromitophagy, and the mitochondrial protease system (Figure 2).

### Mitochondrial morphology control

Mitochondria, as highly dynamic organelles, participate in calcium networks and apoptosis, which are coupled to molecular patterns signaling, amino acid and lipid metabolism, and cell death. Thus, the maintenance of mitochondrial integrity and homeostasis is critical, which is accomplished through continuous fusion and fission. The process by which two mitochondria fuse into one is known as mitochondrial fusion. Because of the double membranes, mitochondrial fusion includes both outer and inner membrane fusion. Three large dynamin-related GTP-hydrolyzing enzymes, mitofusin 1 (MFN1), mitofusin 2 (MFN2), and optic atrophy 1 (OPA1) are involved in the fusion process (Bertholet et al., 2016; Chan, 2020). In particular, MFN1 and MFN2 are localized on the outer mitochondrial membrane (OMM) and are required for outer membrane fusion (Santel and Fuller, 2001; Rojo et al., 2002; Koshiba et al., 2004). Trans interactions between mitofusin are commonly accepted to mediate the tethering of mitochondria during the fusion process because they are present on opposing mitochondrial membranes and form homo-oligomeric and heterooligomeric complexes for fusion. Models of outer membrane fusion have been proposed. The crystal structures of the MFN1 suggest that an intermolecular interface of the globular GTPase domains modulates membrane tethering (Cao et al., 2017; Yan et al., 2018), whereas another model indicates that the C-terminal domain is also needed (Koshiba et al., 2004; Franco et al., 2016). It has recently been suggested that mitochondrial fusion tethers outer membranes through nucleotide-dependent dimerization (Qi et al., 2016; Cao et al., 2017). Following outer membrane fusion, OPA1 mediates mitochondrial inner membrane fusion. OPA1 is found in two topologically distinct isoforms in different tissues due to alternative splicing and proteolytic processing by mitochondrial proteases OMA1 and YME1L (Xiao et al., 2014; Wai et al., 2015; Anderson et al., 2020). Long-form OPA1 (L-OPA1) and cardiolipin are sufficient to facilitate membrane fusion, and loss of OMA1 delays neurodegeneration by preventing stress-induced OPA1 cleavage processing in mitochondria (Korwitz et al., 2016; Ban et al., 2017). In the OPA1-null cells, mitochondria could only show mitochondrial outer membrane fusion but never progress to inner membrane fusion. In this case, mitochondria appear to fission (Song et al., 2009; Mishra et al., 2014).

Fission is undeniably important for mitochondrial division and quality control, and dynamic-related protein 1 (Drp1) plays a key role in this process. Three Drp1 receptors, mitochondrial fission factor (MFF), mitochondrial dynamics proteins 49 (MiD49), and mitochondrial dynamics proteins 51 (MiD51), are all involved in recruiting Drp1 from the cytoplasm to the OMM. A fission defect similar to Drp1 depletion is generated by the loss of any of the receptors, which causes the mitochondria to elongate noticeably (Losón et al., 2013; Osellame et al., 2016; Otera et al., 2016). Fission 1 (Fis1), another OMM-located protein, has also been shown to recruit Drp1. Overexpression of Fis1 in cells promotes mitochondrial fragmentation, however, deletion of the *FIS1* gene has no effect on mitochondrial morphology or Drp1 recruitment to mitochondria (Yoon et al., 2003; Stojanovski et al., 2004; Otera et al., 2010). Drp1 undergoes structural changes after being recruited to mitochondria, constricting the mitochondrial tubule and inducing mitochondrial fission. The cryo-EM studies indicate that cardiolipin, a lipid enriched in mitochondrial membranes, can modulate the Drp1 structure and thus activate the fission process (Figure 2A; Francy et al., 2017).

### Macromitophagy (Mitophagy)

Autophagy is an important quality control system in the nervous system. In mammals, three different types of autophagy processes have been described: macroautophagy, microautophagy, and chaperone-mediated autophagy (CMA). The primary mechanism of MQC in cells is the macroautophagic degradation of mitochondria, or mitophagy, which is necessary for basal mitochondrial turnover.

In the process of mitophagy, dysfunctional mitochondria are first detected, then separated from the mitochondrial network, and recruited by the mitophagosome. The mitophagosome structures are formed in the absence of the ATG8 family proteins, which are classified as microtubule-associated protein light chain 3 (LC3, including LC3A, LC3B, and LC3C) and GABARAP (GABARAP, GABARAP-L1, and GABARAP-L2) subfamilies. Fusion of mitophagosome with lysosomes for degradation is necessary for the last stage of the elimination of damaged mitochondria (Tsuboyama et al., 2016; Onishi et al., 2021). Four major mitophagy pathways include serine/threonine-protein kinase PINK1 (PINK1)/E3 ubiquitin-protein ligase parkin (Parkin)-mediated mitophagy, Bcl-2/adenovirus E1B 19 kDa interacting protein 3 (BNIP3)/NIP3-like protein X (NIX)-regulated mitophagy, FUN14 domain containing 1 (FUNDC1)-mediated mitophagy, and lipid-related pathways (Figure 2B). To trigger the degradation process, members of the ATG8 family should interact with all four pathways.

PINK1/Parkin-mediated mitophagy is the most well-known pathway. PINK1 is found on the IMM of normal mitochondria and is rapidly degraded by the mitochondrial membrane peptidase and presenilin-associated rhomboid-like protease (PARL) (Mokranjac and Neupert, 2007; Meissner et al., 2011). As a result, PINK1 remains at a low level under healthy conditions. However, when the inner membrane potential is depolarized, PINK1 moves to the OMM instead of IMM to form a dimer and is auto-phosphorylated at Ser228 and Ser402 residues (Okatsu et al., 2012). After being phosphorylated at Ser65 in its ubiquitin-like (Ubl) domain and activated by PINK1, Parkin, one of the E3 ubiquitin ligases, ubiquitinates its substrates like mitofusin (Kondapalli et al., 2012; Shiba-Fukushima et al., 2012; Koyano et al., 2014; Onishi et al., 2021). The autophagy adaptors, such as OPTN and NDP52, are phosphorylated by TANK-binding kinase 1 (TBK1), which recognizes these poly-ubiquitin chains, and binds with autophagy-related ATG8 family proteins via LIR motif, leading to mitophagy (Narendra et al., 2010; Kane et al., 2014; Kazlauskaite et al., 2014; Heo et al., 2015; Okatsu et al., 2015; Ordureau et al., 2015).

Unlike PINK1/Parkin, the BNIP3/NIX pathway is activated independently of changes in mitochondrial membrane potential (Rikka et al., 2011). In the normal state, BNIP3 is typically expressed as an inactive monomer in the cytoplasm, but under the hypoxia condition, BNIP3 is up-regulated, homodimerized, and anchored to the OMM by its C-terminal domain, while simultaneously exposing its N-terminal domain to the cytoplasm (Ray et al., 2000; Kubli et al., 2008; Hanna et al., 2012). At the N-terminal domain of BNIP3, the LC3-interacting region (LIR) motif recognizes and binds LC3, and mutations in the LIR motif prevent the contact with LC3, resulting in mitophagy abnormalities. Besides, phosphorylation at Ser17 and Ser24 near the LIR motif of BLIP3 is also important for BNIP3-LC3 interactions (Zhu et al., 2013). NIX is homology to BNIP3 and contains an LIR motif binding to ATG8 family members LC3A, LC3B, GABARAP, GABARAP-L1, and GABARAP-L2 among others (Hamacher-Brady et al., 2007; Sandoval et al., 2008; Novak et al., 2010). Ser34 and Ser35 of NIX, two serine residues close to the LIR motif, are phosphorylated similarly to BNIP3 in order to stabilize NIX-LC3 interactions and induce mitophagy (Rogov et al., 2017).

Additionally, the FUN14 domain containing 1 (FUNDC1) is also an OMM protein that has an LIR motif. It contains a characteristic LIR motif close to the N-terminus and three transmembrane domains (Liu et al., 2012). Phosphorylation and dephosphorylation on residues Ser13 and Tyr18 near the LIR motif of FUNDC1 regulate the process of mitophagy. Under hypoxia conditions, FUNDC1 interacts with LC3 via phosphoglycerate mutase family member 5 phosphatase (PGAM5) dephosphorylation at Ser13, and FUNDC1 phosphorylated by CK2 can reverse the effect of PGAM5 on mitophagy activation (Chen G. et al., 2014). In addition, SRC tyrosine kinase mediates the phosphorylation of Tyr18 to negatively regulate the FUNDC1-LC3 interactions field (Chen et al., 2016). Moreover, the phosphorylation of Ser17 in FUNDC1 by ULK1 enhanced the interaction between FUNDC1 and LC3, which could promote the mitophagy process (Wu et al., 2014).

Likewise, lipids including cardiolipin, cholesterol, and fatty acids have a role in the regulation of mitophagy. Among them, cardiolipin is a mitochondria-specific phospholipid located in IMM and is involved in receptor-mediated mitophagy in cells (Chu et al., 2013). When oxidized, cardiolipin is redistributed and translocated from IMM to OMM in damaged mitochondria and recognized by LC3. This process is coordinated by a hexameric intermembrane space protein, NDPK-D. The knockdown of endogenous NDPK-D decreases cardiolipin externalization and mitochondrial degradation (Kagan et al., 2016). Meanwhile, fatty acids could support the stability of PINK1 and translocation of the Parkin protein, participating in the regulation of mitophagy in the presence of PINK1. In the meantime, it has been shown that cholesterol has a dual role in PINK1/Parkin-mediated mitophagy (Roca-Agujetas et al., 2021).

### Micromitophagy

Micromitophagy, which is defined as mitochondrial degradation independent of mitophagosomes, is another MQC mechanism that ensures mitochondrial homeostasis (Figure 2C; Wang et al., 2022). Under oxidative stress, mitochondria can generate mitochondrial-derived vesicles (MDVs) to proceed micromitophagy. In mammalian cells, MDVs formation is required OMM protein TOM20, or PINK1, Parkin, and soluble N-ethylmaleimide-sensitive factor attachment protein receptors (SNAREs). Recognition and initiation of micromitophagy are different from the mechanism mediated by canonical autophagy regulators such as ATG5 or LC3 (Soubannier et al., 2012; McLelland et al., 2014, 2016; König et al., 2021). Internalization of MDVs into the lysosomal lumen for degradation occurs following the formation of MDVs (Soubannier et al., 2012; Wang et al., 2022). An alternative micromitophagy mechanism known as spermatogenesis-associated protein 18 (SPATA18; also called Mieap)-induced accumulation of lysosome-like organelles within mitochondria (MALM) is characterized by the transfer of lysosomal proteins into mitochondria to destroy the oxidized mitochondrial protein. This process varies from MDVs uptaking microautophagic payloads into lysosomes (Kitamura et al., 2011; Nakamura et al., 2012). Under mitochondrial stress, Mieap is up-regulated and works with BNIP3 to promote MALM (Nakamura et al., 2012). When mitochondria are severely damaged or MALM is inhibited, Mieap-induced vacuoles (MIVs) uptake the entire damaged mitochondria into the lysosome for decay (Kitamura et al., 2011; Miyamoto et al., 2011). Although the micromitophagy processes have been characterized in yeast and some mammalian cells such as hepatocytes, its role in brain and brain-related diseases need to be further investigated (Kissová et al., 2007; Bhatia-Kiššová and Camougrand, 2010; Lemasters and Zhong, 2018).

### Mitochondrial protease system

Mitochondrial proteome is encoded by both nuclear- and mitochondrial-genome. The fidelity and synchronization of these two protein synthesis systems are essential for ATP production and mitochondrial function. The nuclear-encoded proteins are synthesized by cytosolic ribosomes and imported into mitochondria by elaborate machinery embedded in the outer and inner mitochondrial membrane. During this coordination, the production of mitochondrial encoded proteins is regulated by the levels of imported proteins, preventing the redundant unassembled subunits (Ott et al., 2016; Priesnitz and Becker, 2018). The mitochondrial proteases could remove the unassembled proteins of the IMM. Furthermore, upon being imported into mitochondria, proteins synthesized by cytosolic ribosomes are monitored by mitochondrial proteases. Mitochondria have various mitoproteases, which can be divided into processing peptidases, ATP-dependent peptidases, and other mitochondrial peptidases (Deshwal et al., 2020). Among them, mitochondrial processing peptidases remove sorting signals from newly imported nuclear-encoded proteins, which is required for the maturation of many mitochondrial proteins (Mossmann et al., 2012; Poveda-Huertes et al., 2017; Deshwal et al., 2020). The mitochondrial processing protease MPP, for example, cleaves off mitochondrial targeting sequences (MTSs) in the matrix (Mach et al., 2013). Meanwhile, the inner membrane protease (IMMP) or ATP23 promotes the maturation of some proteins into the intermembrane space (IMS) (Weckbecker et al., 2012).

Another type of MQC-related protease is ATP-dependent proteases, which are the core consistency of the mitochondrial proteolytic system, activating in all mitochondrial compartments. The LONP1, caseinolytic mitochondrial matrix peptidase (CLPXP), m-AAA protease, and i-AAA protease are the four ATP-dependent proteases. LONP1 regulates mitochondrial oxidative phosphorylation (OXPHOS) via degrading damaged aconitase, and enzyme of the Krebs cycle in the mitochondrial matrix (Figure 2D; Bota and Davies, 2016). Besides, LONP1 modulates mitochondrial gene expression and some protein maturation (Lagouge et al., 2015; Zurita Rendón and Shoubridge, 2018). LONP1 also facilitates degrade COX4-1 to promote the assembly of the terminal electron transport chain (ETC) enzyme cytochrome c oxidase (Sepuri et al., 2017). Because of the critical role of LONP1 in mitochondrial functions, abnormal LONP1 causes a variety of diseases in humans. CLPXP, other than LONP1, is also reported to participate in the degradation of damaged OXPHOS complex I and II subunits (Seo et al., 2016). Meanwhile, CLPXP regulates gene expression by controlling the mitochondrial RNA (mtRNA) stability (Matsushima et al., 2017).

The m-AAA and i-AAA proteases are mitochondrial membrane-localized proteases (Figure 2D). They are necessary for the proteolysis of misfolded or damaged proteins and some IMM proteins, which helps to maintain the stability of mitochondria. In mammalian mitochondria, the m-AAA protease is composed of either an AFG3L2 homohexamer or an AFG3L2 and SPG7 heterohexamer. The hexameric AAA protease p97 (VCP) has a critical role in the degradation of outer mitochondrial membrane proteins, such as MFN1 (Xu et al., 2011), and the i-AAA protease YME1L removes translocase of the inner membrane 17A protein (TIM17A) to reduce protein import into mitochondria under stress and also regulates mitochondrial lipid composition by degradation of some lipid transfer proteins that shuttle phospholipids across the intermembrane space between the OMM and the IMM (Rainbolt et al., 2013; Saita et al., 2018). Additionally, the IMM proteases also regulate mitochondrial morphology by cleaving OPA1. Deletion of YME1L in the nervous system causes spinal cord axon degeneration in mice. Ablation of metalloproteinase OMA1, which is located on IMM, prevents neurodegeneration in YME1L-mutant mice, demonstrating the role of proteolytic processing in regulating mitochondrial function and physiology in the brain (Sprenger et al., 2019). In addition, the OMM-located ATPase family AAA domain-containing 1 (ATAD1) may promote the extraction and degradation of mislocalized tail-anchored (TA) proteins to preserve mitochondrial integrity, performing a crucial role in the regulation of synaptic activities in neurons (Chen Y. C. et al., 2014; Han et al., 2020).

## Aberrant mitochondrial quality control and brain disorders

Mitochondrial dysfunction impairs mitochondrial respiration, energy generation, mitochondrial oxidative stress, and cell death (Martin, 2010; Yin et al., 2016; Stefanatos and Sanz, 2018; Bader and Winklhofer, 2020). Prior studies have largely focused on how abnormal MQC contributes to various neurological diseases. As an essential part of the nervous system, brain abnormality has always been linked to mitochondrial dysfunction. Here, we briefly outline some MQC disruption-related brain disorders and set out to summarize identified proteins in pathological pathways.

### Neurodegenerative diseases

Neurodegenerative diseases, including Alzheimer’s disease (AD), Parkinson’s disease (PD), Huntington’s disease (HD), and amyotrophic lateral sclerosis (ALS), are characterized by the loss of selective neuron subtypes in the CNS. It has been demonstrated that the aberrant MQC plays a significant role in the progression of these diseases. If we take AD as an example, the mitochondrial fission and fusion proteins are disrupted in the hippocampus in various AD animal models and AD patients. The fission protein Fis1 is up-regulated, while fusion proteins MFN1, MFN2, and OPA1 are down-regulated (Wang et al., 2009). Moreover, Drp1 phosphorylation at Ser616 is higher in the brains of AD patients (Wang et al., 2009). Even though Parkin-mediated mitophagy was initially characterized in PD, the Parkin protein level has been shown higher and Parkin-mediated mitophagy is ineffective in the brains of AD patients (Ye et al., 2015). In addition, abnormal MQC and dysfunctional mitochondria are associated with chronic inflammation. It is well-known that, activated microglia function as innate immune cells in the CNS. In AD mice and patients, as observed, microglia stimulate phagocytosis, clearance, and degradation to minimize the accumulation of Aβ (Graeber et al., 2011). However, chronic microglia activation leads to the secretion of inflammatory cytokines and further neuronal dysfunction (Jiang et al., 2012). These findings suggest that aberrant MQC contributes to the development of neurodegenerative diseases.

### Ischemic stroke

Ischemic stroke is one of the major diseases that cause death and disability of the nervous system. Although revascularization via reperfusion has led to a reduction in the mortality rate of ischemic stroke, the reperfusion itself also causes additional damage to the brain tissue, which is called ischemia-reperfusion (I/R) injury. It has been suggested that several MQC-related processes contribute to I/R damage. The removal of damaged mitochondria and mitochondrial apoptosis of neuron cells is aided by mitochondrial fission and mitophagy during cerebral I/R injury (Kumar et al., 2016; Zhao et al., 2018b). Overexpression of Sirtuin 3 (Sirt3) can inhibit mitochondrial fission and trigger pro-survival signals in neurons subjected to I/R injury (Zhao et al., 2018a). Furthermore, excessive mitochondrial fission stimulates energy imbalance and mtDNA damage, which worsens brain damage (Yue et al., 2015).

### Epilepsy

Epilepsy is typified by recurrent unprovoked seizures of neurons. Mitochondrial stress and MQC are involved in the pathogenesis of epilepsy. The levels of MQC-related proteins OPA1, MFN2, MFF, and Drp1 are elevated in the mice models of acute maximal electroshock and 6 Hz 44 mA seizure (Cho et al., 2022). MQC-related serine peptidase LONP1 is up-regulated in the mitochondria during status epilepticus (SE), and LONP1 knockdown enhances SE-induced mitochondrial apoptosis in neuron (Kim et al., 2021).

### Mitochondrial quality control-related gene mutation or abnormal expression

Mitochondrial quality control-related gene mutation or abnormal expression is related to brain dysplasia (Table 1). In cultured neurons, over-expression of MFN2 mutation disrupted axonal mitochondrial positioning and promoted axon degeneration (Detmer and Chan, 2007; Misko et al., 2012). Moreover, neuron-specific knockout mice show that MFN2 is required for dendritic outgrowth, axonal projection, and survival (Chen et al., 2003, 2007; Lee et al., 2012; Pham et al., 2012). It has been improved that OPA1 can protect neurons from excitotoxicity (Jahani-Asl et al., 2011; Nguyen et al., 2011; Kushnareva et al., 2013). Some research shows heterozygous *OPA1* mutations both on the GTPase or GED domains lead to a decrease of protein quantity and mice of *Opa1* heterozygous mutations have become autosomal dominant optic atrophy (DOA) models. These DOA mice perform retinal ganglionic cell (RGC) loss or dysfunction and optic nerve dysfunction including axonal degeneration and demyelination (Williams et al., 2011). Moreover, mutations of mitophagy-associated gene *PARK6* (encoded PINK1) or *PARK2* (encoded Parkin) contribute to autosomal recessive juvenile parkinsonism (Lücking et al., 1998; Valente et al., 2001, 2002, 2004).

**TABLE 1:** Mitochondrial quality control gene abnormality-related diseases.

Gene	Protein	Variation/Regulation	Symptom/Disease	References
**Mitochondrial morphology**				
*DNM1L*	Drp1	Heterozygous or *de novo* mutations	Multisystem failure, including microcephaly, optic atrophy, hypoplasia, lactic acidemia epilepsy, epileptic encephalopathy and development delay	Waterham et al., 2007; Vanstone et al., 2016; Verrigni et al., 2019
		Phosphorylation at Ser616	AD, PD	Wang et al., 2009; Han et al., 2020
*OPA1*	OPA1	Autosomal dominant	Vision loss, optic nerve dominant optic atrophy (DOA), and DOA plus, Behr syndrome (BEHRS)	Alexander et al., 2000; Amati-Bonneau et al., 2008; Carelli et al., 2015
		Down-regulated	AD	Wang et al., 2009
*MFN2*	MFN2	Recessive mutation	Canine fetal-onset neuroaxonal dystrophy	Fyfe et al., 2011
		Down-regulated	AD	Wang et al., 2009
*MEF2B*	MEF	Recessive mutation	Delayed childhood development, optic atrophy, seizures, peripheral neuropathy, hypotonia	Shamseldin et al., 2012; Koch et al., 2016
**Mitophagy**				
*PARK6*	PINK1	Mutation	PD	Valente et al., 2001, 2002, 2004
*PARK2*	Parkin	Mutation	PD	Kitada et al., 1998; Lücking et al., 1998
		Up-regulated	AD	Ye et al., 2015
*HDAC6*	HDAC6	Mutation	Chondrodysplasia with platyspondyly, distinctive brachydactyly, hydrocephaly, and microphthalmia (CDP-PBHM), PD	Lee et al., 2010; Simon et al., 2010
**Mitochondrial proteases**				
*LONP1*	LONP1	Mutation	Cerebral, ocular, dental, auricular, and skeletal (CODAS) syndrome	Strauss et al., 2015; Peter et al., 2018
*AFG3L2*	AFG3L2	Dominant mutation	Spinocerebellar ataxia, spastic ataxia (SCA28), optic atrophy 12 (OPA12)	Di Bella et al., 2010; Caporali et al., 2020
		Recessive mutation	Spastic ataxia 5 (SPAX5)	Pierson et al., 2011
*YME1L*	YME1L1	Mutation	Mitochondriopathy with optic atrophy	Hartmann et al., 2016

## Potential of mitochondrial quality control-related targets for brain disorders

Mitochondrial quality control-related protein inhibitors and agonists have been recently shown to suppress pathological processes by regulating mitochondrial functions, including nervous system diseases, cardiovascular diseases, metabolic diseases, and cancer. In light of this, researchers are looking into whether MQC-related proteins can be drug targets for these diseases. For instance, a small molecule SIRT1 activator, SRT-1720, markedly improves renal tubular pathology and overall renal function in adult mice following I/R via regulating mitophagy (Fan et al., 2013). Mdivi-1, the inhibitor of Drp1, can induce apoptosis of hepatocellular carcinoma cells, suggesting a new approach of targeting MQC in cancer treatment (Akita et al., 2014; Wang et al., 2015; Lin et al., 2020). Thus, MQC-related proteins may become possible targets for disease treatment. Likewise, MQC is crucial for preserving healthy mitochondria and preventing the pathological effects of dysfunctional mitochondria in the brain. MQC-related targets, accordingly, represent potential future therapeutic strategies for brain diseases.

The mitochondrial morphology-associated protein Drp1 is highly expressed in the brain, implying that it is an important component in the brain. It has been demonstrated to modulate both the death of the neuronal cell and the survival of post-mitotic neurons (Cribbs and Strack, 2007; Kageyama et al., 2012). The inhibitor of Drp1, Mdvi-1, provides neuroprotection *in vitro* and *in vivo* (Cassidy-Stone et al., 2008; Park et al., 2011; Grohm et al., 2012). Pre- and post-treated AD or PD cells with Drp1 inhibitor Mdivi-1 show decreasing interaction between Drp1 and phosphorylated tau, reducing Aβ or α-syn aggregates, suppressing mitochondrial dysfunction, and maintaining cell viability, mitochondrial dynamics, mitochondrial biogenesis, and synaptic activity, indicating neuroprotection effects (Manczak and Reddy, 2012; Reddy et al., 2017; Wang et al., 2017). Meanwhile, inhibition of mitochondrial fission also suppresses the progress of ALS. The SOD1G93A mouse model is used for preclinical testing of treatments for ALS (Gurney et al., 1994). P100, which inhibits the interaction of Drp1 and Fis1, improves the mitochondrial structure and function by reducing oxidative stress in this model. Besides, P110 treatment also suppresses mitochondrial dysfunction in motor neurons and patient-derived fibroblasts, suggesting that Drp1 may be a drug target in ALS therapy strategy (Joshi et al., 2018). Furthermore, treatment with mitochondrial-targeted donor AP39 can transfer mitochondrial fission to fusion by increasing OPA1 and MFN1 levels and decreasing Fis1 levels in early-onset AD model APP/PS1 neurons and transgenic mice (Cassidy-Stone et al., 2008; Park et al., 2011; Grohm et al., 2012).

Mitophagy-associated proteins and mitochondrial proteases are also potential drug targets for brain diseases. Compound BC1464, which disrupts the FBXO7/PINK1 interaction, can rescue mitophagy and provides neuroprotection in PD models (Liu et al., 2020). The ATPase inhibitor, KUS121, improves the average readable letter counts, visual field scores, and retinal sensitivities of all nine patients with acute central retinal artery occlusion (CRAO) in phase I/II clinical trial (Hanako Ohashi et al., 2020). In the meanwhile, KUS121 shows the effect of preventing retinal ganglion cell death in animal models of glaucoma (Nakano et al., 2016).

As can be seen, these studies indicate that MQC-associated proteins are suitable as therapeutic pharmaceutical targets for brain disorders (Table 2).

**TABLE 2:** Mitochondrial quality control (MQC)-associated proteins and therapeutic agents in brain diseases.^a^

Target	PDB code^b^	Treatment	Indications/Condition	Highest phase	References
**Mitochondrial morphology**					
Drp1	4BEJ, O00429	Mdivi-1	AD, HD, PD	Biological testing	Cui et al., 2010; Manczak and Reddy, 2012; Fröhlich et al., 2013; Rappold et al., 2014; Bido et al., 2017; Reddy et al., 2017; Wang et al., 2017
		P110/P110-TAT	ALS, HD	Preclinical	Joshi et al., 2018
		Lenti-Drp1-S579A	Neurodegenerative diseases	Biological testing	–
Fis1	1NZN, 1PC2, Q9Y3D6	P110	ALS, PD, HD	Preclinical	Suzuki et al., 2003; Dohm et al., 2004; Joshi et al., 2018
OPA1	6JTG, O60313	STK-002	Dominant optic atrophy	Preclinical	Yu et al., 2020
		VP-002	Ocular genetic disorders	Preclinical	–
		NFS-05 rAAV2-OPA1	Optic neuropathy	Preclinical	–
		rAAV2-hOPA1	Optic neuropathy	Biological testing	–
		pAAV2-OPA1-ND4	Leber hereditary optic neuropathy	Biological testing	–
MFN1	5GNU, 5YEW, 5GOE, Q8IWA4^c^	MiM-111	ALS, HD, PD	Biological testing	Qi et al., 2016; Cao et al., 2017; Yan et al., 2018
		Chimera-C Regeneurin-C	AD, PD, HD	Biological testing	–
MFN2	6JFK, 6JFM, O95140^c^	MiM-111	ALS, HD, PD	Biological testing	Li et al., 2019
		Chimera B-A/l/, Mfn2-367-384Gly-TAT/TAT-367-384Gly	Charcot-Marie-Tooth disease, type 2A	Preclinical	–
		MASM-7	Neurological disorders	Biological testing	–
		Chimera-C Regeneurin-C Mitolityn-4 Regeneurin-C/O	AD, PD, HD	Biological testing	–
MiD49	5WP9, Q96C03^c^	–	–	–	Kalia et al., 2018
MiD51	4NXT, Q9NQG6^c^	–	–	–	Richter et al., 2014
MFF	Q9GZY8^c^	–	–	–	–
**Mitophagy**					
PINK1	Q9BXM7^c^	MTK-115	HD	Biological testing	–
		BC1464	PD	Biological testing	Liu et al., 2020
		MTK-0034/0030/0043	PD	Biological testing	–
Parkin	5N38, O60260^c^	–	–	–	Kumar et al., 2017
FUNDC1	2N9X, 5GMV, Q8IVP5^c^	–	–	–	Kuang et al., 2016; Lv et al., 2017
ULK1	6QAS, O75385^c^	VMY-BC-1	Brain cancer	Preclinical	Chaikuad et al., 2019
		BL-918	PD	Preclinical	–
TBK1	6NT9, Q9UHD2^c^	GSK-8612	Neurological disorders	Biological testing	Zhang et al., 2019
BNIP3	2J5D (Bocharov et al., 2007), Q12983^c^	–	–	–	Bocharov et al., 2007
NIX	O60238^c^	–	–	–	–
PGAM5	5MUF, Q96HS1^c^	–	–	–	Chaikuad et al., 2017
Beclin 1	7BL1, Q14457^c^	–	–	–	Tremel et al., 2021
Beclin 2	5K7B, 5K9L, A8MW95^c^	–	–	–	Koentjoro et al., 2017
SIRT1	5BTR, Q96EB6^c^	SRTAW-04	Neurodegeneration	Preclinical	Cao et al., 2015
**Mitochondrial proteases**					
p97 (VCP)	7RLF	KUS-121	Central retinal artery occlusion (CRAO), PD, Glaucoma Retinal degeneration Stroke, ischemic	Phase I/II	Hasegawa et al., 2016, 2020; Hanako Ohashi et al., 2020; Caffrey et al., 2021
		KUS-187	Ocular genetic disorders	Priclinical	–
LONP1	7OXO	BT-317	Glioblastoma multiforme therapy	Biological testing	Mohammed et al., 2022
AFG3L2	2LNA, 6NYY, Q9Y4W6^c^	–	–	–	Ramelot et al., 2013; Puchades et al., 2019
YME1L1	Q96TA2^c^	–	–	–	–

^a^Part of the data from *Cortellis Drug Discovery Intelligence* database.

^b^For some target proteins, there are a considerable number of PDB codes, and only some of the results are shown here.

^c^AlphaFoldDB of the target proteins.

## Conclusion and perspective

In this review, we discuss the physiological roles of mitochondria and the MQC mechanism in the brain. MQC not only plays a vital role in maintaining mitochondrial morphology and functioning, but also participates in the pathological progression of a range of brain illnesses. Regulation of MQC through the pharmacological intervention of mitochondrial morphology, mitophagy, or the activity of mitochondrial proteases is emerging as a strategy for the treatment of mitochondrial-associated brain disorders.

Although MQC regulation can improve the process of brain disease, only a few regulators of MQC-related proteins have been identified as novel therapeutic targets or used in preclinical research (Table 2). Thus, there is still a lack of effective regulators, and developing targeted drugs is incredibly challenging. Because of the massive data sets available for drug candidates, computer-aided drug design (CADD) offers new approaches to efficacy and safety evaluations of drug candidates based on big data modeling, artificial intelligence modeling, and molecular docking (Zhu, 2020). This targeted drug development is dependent on the protein structure of the target (Yang et al., 2021). Some MQC-related essential proteins’ structures have been analyzed or predicted as structural biology and structure prediction methods have advanced, but PDB structures of full-length proteins under different conditions, as well as functional complexes, require further investigation (Table 2).

Finally, as we reviewed, although it is of bright prospects to develop MQC-related proteins as novel drug targets for brain disorders, the treatment of which still has a long way to go. Meanwhile, when some MQC-regulated drugs are in clinical trials, larger-scale clinical studies will be required to verify the safety and effectiveness of the drugs. Hence, in the future, more in-depth understanding of MQC would give rise to the development in the treatment of neurological related diseases, upon which more innovative therapeutic options will come to fruit.

## Author contributions

HY and XS conceived the topic for this review. XS, PS, and HZ prepared the figures and tables. All authors listed wrote the manuscript and approved the submitted version.

## References

[B1] Akita M. Suzuki-Karasaki M. Fujiwara K. Nakagawa C. Soma M. Yoshida Y. (2014). Mitochondrial division inhibitor-1 induces mitochondrial hyperfusion and sensitizes human cancer cells to TRAIL-induced apoptosis. *Int. J. Oncol.* 45 1901–1912. 10.3892/ijo.2014.2608 25174275

[B2] Alexander C. Votruba M. Pesch U. E. Thiselton D. L. Mayer S. Moore A. (2000). OPA1, encoding a dynamin-related GTPase, is mutated in autosomal dominant optic atrophy linked to chromosome 3q28. *Nat. Genet.* 26 211–215. 10.1038/79944 11017080

[B3] Amati-Bonneau P. Valentino M. L. Reynier P. Gallardo M. E. Bornstein B. Boissière A. (2008). OPA1 mutations induce mitochondrial DNA instability and optic atrophy ‘plus’ phenotypes. *Brain* 131 338–351. 10.1093/brain/awm298 18158317

[B4] Ambekar T. Pawar J. Rathod R. Patel M. Fernandes V. Kumar R. (2021). Mitochondrial quality control: Epigenetic signatures and therapeutic strategies. *Neurochem. Int.* 148:105095. 10.1016/j.neuint.2021.105095 34111479

[B5] Anderson C. J. Kahl A. Fruitman H. Qian L. Zhou P. Manfredi G. (2020). Prohibitin levels regulate OMA1 activity and turnover in neurons. *Cell Death Differ.* 27 1896–1906. 10.1038/s41418-019-0469-4 31819158 PMC7244729

[B6] Bader V. Winklhofer K. F. (2020). Mitochondria at the interface between neurodegeneration and neuroinflammation. *Semin. Cell Dev. Biol.* 99 163–171. 10.1016/j.semcdb.2019.05.028 31154011

[B7] Ban T. Ishihara T. Kohno H. Saita S. Ichimura A. Maenaka K. (2017). Molecular basis of selective mitochondrial fusion by heterotypic action between OPA1 and cardiolipin. *Nat. Cell Biol.* 19 856–863. 10.1038/ncb3560 28628083

[B8] Bertholet A. M. Delerue T. Millet A. M. Moulis M. F. David C. Daloyau M. (2016). Mitochondrial fusion/fission dynamics in neurodegeneration and neuronal plasticity. *Neurobiol. Dis.* 90 3–19. 10.1016/j.nbd.2015.10.011 26494254

[B9] Bhatia-Kiššová I. Camougrand N. (2010). Mitophagy in yeast: Actors and physiological roles. *FEMS Yeast Res.* 10 1023–1034. 10.1111/j.1567-1364.2010.00659.x 20629757

[B10] Bido S. Soria F. N. Fan R. Z. Bezard E. Tieu K. (2017). Mitochondrial division inhibitor-1 is neuroprotective in the A53T-α-synuclein rat model of Parkinson’s disease. *Sci. Rep.* 7:7495. 10.1038/s41598-017-07181-0 28790323 PMC5548731

[B11] Bocharov E. V. Pustovalova Y. E. Pavlov K. V. Volynsky P. E. Goncharuk M. V. Ermolyuk Y. S. (2007). Unique dimeric structure of BNip3 transmembrane domain suggests membrane permeabilization as a cell death trigger. *J. Biol. Chem.* 282 16256–16266. 10.1074/jbc.M701745200 17412696

[B12] Bock F. J. Tait S. W. G. (2020). Mitochondria as multifaceted regulators of cell death. *Nat. Rev. Mol. Cell Biol.* 21 85–100. 10.1038/s41580-019-0173-8 31636403

[B13] Bota D. A. Davies K. J. (2016). Mitochondrial Lon protease in human disease and aging: Including an etiologic classification of Lon-related diseases and disorders. *Free Radic. Biol. Med.* 100 188–198. 10.1016/j.freeradbiomed.2016.06.031 27387767 PMC5183306

[B14] Buss R. R. Gould T. W. Ma J. Vinsant S. Prevette D. Winseck A. (2006). Neuromuscular development in the absence of programmed cell death: Phenotypic alteration of motoneurons and muscle. *J. Neurosci.* 26 13413–13427. 10.1523/jneurosci.3528-06.2006 17192424 PMC6674711

[B15] Caffrey B. Zhu X. Berezuk A. Tuttle K. Chittori S. Subramaniam S. (2021). AAA+ ATPase p97/VCP mutants and inhibitor binding disrupt inter-domain coupling and subsequent allosteric activation. *J. Biol. Chem.* 297:101187. 10.1016/j.jbc.2021.101187 34520757 PMC8517850

[B16] Cao D. Wang M. Qiu X. Liu D. Jiang H. Yang N. (2015). Structural basis for allosteric, substrate-dependent stimulation of SIRT1 activity by resveratrol. *Genes Dev.* 29 1316–1325. 10.1101/gad.265462.115 26109052 PMC4495401

[B17] Cao Y. L. Meng S. Chen Y. Feng J. X. Gu D. D. Yu B. (2017). MFN1 structures reveal nucleotide-triggered dimerization critical for mitochondrial fusion. *Nature* 542 372–376. 10.1038/nature21077 28114303 PMC5319402

[B18] Caporali L. Magri S. Legati A. Del Dotto V. Tagliavini F. Balistreri F. (2020). ATPase domain AFG3L2 mutations alter OPA1 processing and cause optic neuropathy. *Ann. Neurol.* 88 18–32. 10.1002/ana.25723 32219868 PMC7383914

[B19] Carelli V. Sabatelli M. Carrozzo R. Rizza T. Schimpf S. Wissinger B. (2015). ‘Behr syndrome’ with OPA1 compound heterozygote mutations. *Brain* 138:e321. 10.1093/brain/awu234 25146916 PMC4441076

[B20] Cassidy-Stone A. Chipuk J. E. Ingerman E. Song C. Yoo C. Kuwana T. (2008). Chemical inhibition of the mitochondrial division dynamin reveals its role in Bax/Bak-dependent mitochondrial outer membrane permeabilization. *Dev. Cell* 14 193–204. 10.1016/j.devcel.2007.11.019 18267088 PMC2267902

[B21] Chaikuad A. Filippakopoulos P. Marcsisin S. R. Picaud S. Schröder M. Sekine S. (2017). Structures of PGAM5 provide insight into active site plasticity and multimeric assembly. *Structure* 25 1089–1099.e3. 10.1016/j.str.2017.05.020 28648608 PMC5501728

[B22] Chaikuad A. Koschade S. E. Stolz A. Zivkovic K. Pohl C. Shaid S. (2019). Conservation of structure, function and inhibitor binding in UNC-51-like kinase 1 and 2 (ULK1/2). *Biochem. J.* 476 875–887. 10.1042/bcj20190038 30782972 PMC10275411

[B23] Chan D. C. (2020). Mitochondrial dynamics and its involvement in disease. *Annu. Rev. Pathol.* 15 235–259. 10.1146/annurev-pathmechdis-012419-032711 31585519

[B24] Chen G. Han Z. Feng D. Chen Y. Chen L. Wu H. (2014). A regulatory signaling loop comprising the PGAM5 phosphatase and CK2 controls receptor-mediated mitophagy. *Mol. Cell* 54 362–377. 10.1016/j.molcel.2014.02.034 24746696

[B25] Chen H. Detmer S. A. Ewald A. J. Griffin E. E. Fraser S. E. Chan D. C. (2003). Mitofusins Mfn1 and Mfn2 coordinately regulate mitochondrial fusion and are essential for embryonic development. *J. Cell Biol.* 160 189–200. 10.1083/jcb.200211046 12527753 PMC2172648

[B26] Chen H. McCaffery J. M. Chan D. C. (2007). Mitochondrial fusion protects against neurodegeneration in the cerebellum. *Cell* 130 548–562. 10.1016/j.cell.2007.06.026 17693261

[B27] Chen M. Chen Z. Wang Y. Tan Z. Zhu C. Li Y. (2016). Mitophagy receptor FUNDC1 regulates mitochondrial dynamics and mitophagy. *Autophagy* 12 689–702. 10.1080/15548627.2016.1151580 27050458 PMC4836026

[B28] Chen Y. C. Umanah G. K. Dephoure N. Andrabi S. A. Gygi S. P. Dawson T. M. (2014). Msp1/ATAD1 maintains mitochondrial function by facilitating the degradation of mislocalized tail-anchored proteins. *EMBO J.* 33 1548–1564. 10.15252/embj.201487943 24843043 PMC4198051

[B29] Cheng X. Geng F. Pan M. Wu X. Zhong Y. Wang C. (2020). Targeting DGAT1 ameliorates glioblastoma by increasing fat catabolism and oxidative stress. *Cell Metab.* 32 229–242.e8. 10.1016/j.cmet.2020.06.002 32559414 PMC7415721

[B30] Cho C. Zeigler M. Mizuno S. Morrison R. S. Totah R. A. Barker-Haliski M. (2022). Reductions in hydrogen sulfide and changes in mitochondrial quality control proteins are evident in the early phases of the corneally kindled mouse model of epilepsy. *Int. J. Mol. Sci.* 23:1434. 10.3390/ijms23031434 35163358 PMC8835945

[B31] Chouchani E. T. Pell V. R. Gaude E. Aksentijević D. Sundier S. Y. Robb E. L. (2014). Ischaemic accumulation of succinate controls reperfusion injury through mitochondrial ROS. *Nature* 515 431–435. 10.1038/nature13909 25383517 PMC4255242

[B32] Chu C. T. Ji J. Dagda R. K. Jiang J. F. Tyurina Y. Y. Kapralov A. A. (2013). Cardiolipin externalization to the outer mitochondrial membrane acts as an elimination signal for mitophagy in neuronal cells. *Nat. Cell Biol.* 15 1197–1205. 10.1038/ncb2837 24036476 PMC3806088

[B33] Cribbs J. T. Strack S. (2007). Reversible phosphorylation of Drp1 by cyclic AMP-dependent protein kinase and calcineurin regulates mitochondrial fission and cell death. *EMBO Rep.* 8 939–944. 10.1038/sj.embor.7401062 17721437 PMC2002551

[B34] Cui M. Tang X. Christian W. V. Yoon Y. Tieu K. (2010). Perturbations in mitochondrial dynamics induced by human mutant PINK1 can be rescued by the mitochondrial division inhibitor mdivi-1. *J. Biol. Chem.* 285 11740–11752. 10.1074/jbc.M109.066662 20164189 PMC2857048

[B35] Deshwal S. Fiedler K. U. Langer T. (2020). Mitochondrial proteases: Multifaceted regulators of mitochondrial plasticity. *Annu. Rev. Biochem.* 89 501–528. 10.1146/annurev-biochem-062917-012739 32075415

[B36] Detmer S. A. Chan D. C. (2007). Complementation between mouse Mfn1 and Mfn2 protects mitochondrial fusion defects caused by CMT2A disease mutations. *J. Cell Biol.* 176 405–414. 10.1083/jcb.200611080 17296794 PMC2063976

[B37] Devine M. J. Kittler J. T. (2018). Mitochondria at the neuronal presynapse in health and disease. *Nat. Rev. Neurosci.* 19 63–80. 10.1038/nrn.2017.170 29348666

[B38] Di Bella D. Lazzaro F. Brusco A. Plumari M. Battaglia G. Pastore A. (2010). Mutations in the mitochondrial protease gene AFG3L2 cause dominant hereditary ataxia SCA28. *Nat. Genet.* 42 313–321. 10.1038/ng.544 20208537

[B39] Dohm J. A. Lee S. J. Hardwick J. M. Hill R. B. Gittis A. G. (2004). Cytosolic domain of the human mitochondrial fission protein fis1 adopts a TPR fold. *Proteins* 54 153–156. 10.1002/prot.10524 14705031 PMC3047745

[B40] Fan H. Yang H. C. You L. Wang Y. Y. He W. J. Hao C. M. (2013). The histone deacetylase, SIRT1, contributes to the resistance of young mice to ischemia/reperfusion-induced acute kidney injury. *Kidney Int.* 83 404–413. 10.1038/ki.2012.394 23302720

[B41] Franco A. Kitsis R. N. Fleischer J. A. Gavathiotis E. Kornfeld O. S. Gong G. (2016). Correcting mitochondrial fusion by manipulating mitofusin conformations. *Nature* 540 74–79. 10.1038/nature20156 27775718 PMC5315023

[B42] Francy C. A. Clinton R. W. Fröhlich C. Murphy C. Mears J. A. (2017). Cryo-EM studies of Drp1 reveal cardiolipin interactions that activate the helical oligomer. *Sci. Rep.* 7:10744. 10.1038/s41598-017-11008-3 28878368 PMC5587723

[B43] Fricker M. Tolkovsky A. M. Borutaite V. Coleman M. Brown G. C. (2018). Neuronal cell death. *Physiol. Rev.* 98 813–880. 10.1152/physrev.00011.2017 29488822 PMC5966715

[B44] Fröhlich C. Grabiger S. Schwefel D. Faelber K. Rosenbaum E. Mears J. (2013). Structural insights into oligomerization and mitochondrial remodelling of dynamin 1-like protein. *EMBO J.* 32 1280–1292. 10.1038/emboj.2013.74 23584531 PMC3642683

[B45] Fyfe J. C. Al-Tamimi R. A. Liu J. Schäffer A. A. Agarwala R. Henthorn P. S. (2011). A novel mitofusin 2 mutation causes canine fetal-onset neuroaxonal dystrophy. *Neurogenetics* 12 223–232. 10.1007/s10048-011-0285-6 21643798 PMC3165057

[B46] Gibbs K. L. Greensmith L. Schiavo G. (2015). Regulation of axonal transport by protein kinases. *Trends Biochem. Sci.* 40 597–610. 10.1016/j.tibs.2015.08.003 26410600

[B47] Graeber M. B. Li W. Rodriguez M. L. (2011). Role of microglia in CNS inflammation. *FEBS Lett.* 585 3798–3805. 10.1016/j.febslet.2011.08.033 21889505

[B48] Grohm J. Kim S. W. Mamrak U. Tobaben S. Cassidy-Stone A. Nunnari J. (2012). Inhibition of Drp1 provides neuroprotection in vitro and in vivo. *Cell Death Differ.* 19 1446–1458. 10.1038/cdd.2012.18 22388349 PMC3422469

[B49] Gu C. Wang F. Zhang Y. T. Wei S. Z. Liu J. Y. Sun H. Y. (2021). Microglial MT1 activation inhibits LPS-induced neuroinflammation via regulation of metabolic reprogramming. *Aging Cell* 20:e13375. 10.1111/acel.13375 33964119 PMC8208780

[B50] Guntuku L. Naidu V. G. Yerra V. G. (2016). Mitochondrial dysfunction in gliomas: Pharmacotherapeutic potential of natural compounds. *Curr. Neuropharmacol.* 14 567–583. 10.2174/1570159x14666160121115641 26791479 PMC4981742

[B51] Gurney M. E. Pu H. Chiu A. Y. Dal Canto M. C. Polchow C. Y. Alexander D. D. (1994). Motor neuron degeneration in mice that express a human Cu,Zn superoxide dismutase mutation. *Science* 264 1772–1775. 10.1126/science.8209258 8209258

[B52] Hamacher-Brady A. Brady N. R. Logue S. E. Sayen M. R. Jinno M. Kirshenbaum L. A. (2007). Response to myocardial ischemia/reperfusion injury involves Bnip3 and autophagy. *Cell Death Differ.* 14 146–157. 10.1038/sj.cdd.4401936 16645637

[B53] Han H. Tan J. Wang R. Wan H. He Y. Yan X. (2020). PINK1 phosphorylates Drp1(S616) to regulate mitophagy-independent mitochondrial dynamics. *EMBO Rep.* 21:e48686. 10.15252/embr.201948686 32484300 PMC7403662

[B54] Hanako Ohashi I. Muraoka Y. Hata M. Sumi E. Ikeda T. Nakagawa T. (2020). Safety and effectiveness of a novel neuroprotectant, KUS121, in patients with non-arteritic central retinal artery occlusion: An open-label, non-randomized, first-in-humans, phase 1/2 trial. *PLoS One* 15:e0229068. 10.1371/journal.pone.0229068 32053676 PMC7018138

[B55] Hanna R. A. Quinsay M. N. Orogo A. M. Giang K. Rikka S. Gustafsson Å. B. (2012). Microtubule-associated protein 1 light chain 3 (LC3) interacts with Bnip3 protein to selectively remove endoplasmic reticulum and mitochondria via autophagy. *J. Biol. Chem.* 287 19094–19104. 10.1074/jbc.M111.322933 22505714 PMC3365942

[B56] Harris J. J. Attwell D. (2012). The energetics of CNS white matter. *J. Neurosci.* 32 356–371. 10.1523/JNEUROSCI.3430-11.2012 22219296 PMC3272449

[B57] Harris J. J. Jolivet R. Attwell D. (2012). Synaptic energy use and supply. *Neuron* 75 762–777. 10.1016/j.neuron.2012.08.019 22958818

[B58] Hartmann B. Wai T. Hu H. MacVicar T. Musante L. Fischer-Zirnsak B. (2016). Homozygous YME1L1 mutation causes mitochondriopathy with optic atrophy and mitochondrial network fragmentation. *eLife* 5:e16078. 10.7554/eLife.16078 27495975 PMC4991934

[B59] Hasegawa T. Ikeda H. O. Gotoh N. Iida K. Iwai S. Nakano N. (2020). Effect of VCP modulators on gene expression profiles of retinal ganglion cells in an acute injury mouse model. *Sci. Rep.* 10:4251. 10.1038/s41598-020-61160-6 32144342 PMC7060332

[B60] Hasegawa T. Muraoka Y. Ikeda H. O. Tsuruyama T. Kondo M. Terasaki H. (2016). Neuoroprotective efficacies by KUS121, a VCP modulator, on animal models of retinal degeneration. *Sci. Rep.* 6:31184. 10.1038/srep31184 27503804 PMC4977562

[B61] Heo J. M. Ordureau A. Paulo J. A. Rinehart J. Harper J. W. (2015). The PINK1-PARKIN mitochondrial ubiquitylation pathway drives a program of OPTN/NDP52 recruitment and TBK1 activation to promote mitophagy. *Mol. Cell* 60 7–20. 10.1016/j.molcel.2015.08.016 26365381 PMC4592482

[B62] Iranmanesh Y. Jiang B. Favour O. C. Dou Z. Wu J. Li J. (2021). Mitochondria’s role in the maintenance of cancer stem cells in glioblastoma. *Front. Oncol.* 11:582694. 10.3389/fonc.2021.582694 33692947 PMC7937970

[B63] Islam M. T. (2017). Oxidative stress and mitochondrial dysfunction-linked neurodegenerative disorders. *Neurol. Res.* 39 73–82. 10.1080/01616412.2016.1251711 27809706

[B64] Jahani-Asl A. Pilon-Larose K. Xu W. MacLaurin J. G. Park D. S. McBride H. M. (2011). The mitochondrial inner membrane GTPase, optic atrophy 1 (Opa1), restores mitochondrial morphology and promotes neuronal survival following excitotoxicity. *J. Biol. Chem.* 286 4772–4782. 10.1074/jbc.M110.167155 21041314 PMC3039390

[B65] Jiang T. Yu J. T. Tan L. (2012). Novel disease-modifying therapies for Alzheimer’s disease. *J. Alzheimers Dis.* 31 475–492. 10.3233/jad-2012-120640 22669013

[B66] Joshi A. U. Saw N. L. Vogel H. Cunnigham A. D. Shamloo M. Mochly-Rosen D. (2018). Inhibition of Drp1/Fis1 interaction slows progression of amyotrophic lateral sclerosis. *EMBO Mol. Med.* 10:e8166. 10.15252/emmm.201708166 29335339 PMC5840540

[B67] Kagan V. E. Jiang J. Huang Z. Tyurina Y. Y. Desbourdes C. Cottet-Rousselle C. (2016). NDPK-D (NM23-H4)-mediated externalization of cardiolipin enables elimination of depolarized mitochondria by mitophagy. *Cell Death Differ.* 23 1140–1151. 10.1038/cdd.2015.160 26742431 PMC4946882

[B68] Kageyama Y. Zhang Z. Roda R. Fukaya M. Wakabayashi J. Wakabayashi N. (2012). Mitochondrial division ensures the survival of postmitotic neurons by suppressing oxidative damage. *J. Cell Biol.* 197 535–551. 10.1083/jcb.201110034 22564413 PMC3352955

[B69] Kalia R. Wang R. Y. Yusuf A. Thomas P. V. Agard D. A. Shaw J. M. (2018). Structural basis of mitochondrial receptor binding and constriction by DRP1. *Nature* 558 401–405. 10.1038/s41586-018-0211-2 29899447 PMC6120343

[B70] Kalpage H. A. Bazylianska V. Recanati M. A. Fite A. Liu J. Wan J. (2019). Tissue-specific regulation of cytochrome c by post-translational modifications: Respiration, the mitochondrial membrane potential, ROS, and apoptosis. *FASEB J.* 33 1540–1553. 10.1096/fj.201801417R 30222078 PMC6338631

[B71] Kane L. A. Lazarou M. Fogel A. I. Li Y. Yamano K. Sarraf S. A. (2014). PINK1 phosphorylates ubiquitin to activate Parkin E3 ubiquitin ligase activity. *J. Cell Biol.* 205 143–153. 10.1083/jcb.201402104 24751536 PMC4003245

[B72] Kazlauskaite A. Kondapalli C. Gourlay R. Campbell D. G. Ritorto M. S. Hofmann K. (2014). Parkin is activated by PINK1-dependent phosphorylation of ubiquitin at Ser65. *Biochem. J.* 460 127–139. 10.1042/bj20140334 24660806 PMC4000136

[B73] Kim J. E. Park H. Kim T. H. Kang T. C. (2021). LONP1 regulates mitochondrial accumulations of HMGB1 and caspase-3 in CA1 and PV neurons following status epilepticus. *Int. J. Mol. Sci.* 22:2275. 10.3390/ijms22052275 33668863 PMC7956547

[B74] Kissová I. Salin B. Schaeffer J. Bhatia S. Manon S. Camougrand N. (2007). Selective and non-selective autophagic degradation of mitochondria in yeast. *Autophagy* 3 329–336. 10.4161/auto.4034 17377488

[B75] Kitada T. Asakawa S. Hattori N. Matsumine H. Yamamura Y. Minoshima S. (1998). Mutations in the parkin gene cause autosomal recessive juvenile parkinsonism. *Nature* 392 605–608. 10.1038/33416 9560156

[B76] Kitamura N. Nakamura Y. Miyamoto Y. Miyamoto T. Kabu K. Yoshida M. (2011). Mieap, a p53-inducible protein, controls mitochondrial quality by repairing or eliminating unhealthy mitochondria. *PLoS One* 6:e16060. 10.1371/journal.pone.0016060 21264228 PMC3022033

[B77] Koch J. Feichtinger R. G. Freisinger P. Pies M. Schrödl F. Iuso A. (2016). Disturbed mitochondrial and peroxisomal dynamics due to loss of MFF causes Leigh-like encephalopathy, optic atrophy and peripheral neuropathy. *J. Med. Genet.* 53 270–278. 10.1136/jmedgenet-2015-103500 26783368

[B78] Koentjoro B. Park J. S. Sue C. M. (2017). Nix restores mitophagy and mitochondrial function to protect against PINK1/Parkin-related Parkinson’s disease. *Sci. Rep.* 7:44373. 10.1038/srep44373 28281653 PMC5345073

[B79] Kondapalli C. Kazlauskaite A. Zhang N. Woodroof H. I. Campbell D. G. Gourlay R. (2012). PINK1 is activated by mitochondrial membrane potential depolarization and stimulates Parkin E3 ligase activity by phosphorylating Serine 65. *Open Biol.* 2:120080. 10.1098/rsob.120080 22724072 PMC3376738

[B80] König T. Nolte H. Aaltonen M. J. Tatsuta T. Krols M. Stroh T. (2021). MIROs and DRP1 drive mitochondrial-derived vesicle biogenesis and promote quality control. *Nat. Cell Biol.* 23 1271–1286. 10.1038/s41556-021-00798-4 34873283

[B81] Korwitz A. Merkwirth C. Richter-Dennerlein R. Tröder S. E. Sprenger H. G. Quirós P. M. (2016). Loss of OMA1 delays neurodegeneration by preventing stress-induced OPA1 processing in mitochondria. *J. Cell Biol.* 212 157–166. 10.1083/jcb.201507022 26783299 PMC4738383

[B82] Koshiba T. Detmer S. A. Kaiser J. T. Chen H. McCaffery J. M. Chan D. C. (2004). Structural basis of mitochondrial tethering by mitofusin complexes. *Science* 305 858–862. 10.1126/science.1099793 15297672

[B83] Koyano F. Okatsu K. Kosako H. Tamura Y. Go E. Kimura M. (2014). Ubiquitin is phosphorylated by PINK1 to activate parkin. *Nature* 510 162–166. 10.1038/nature13392 24784582

[B84] Kuang Y. Ma K. Zhou C. Ding P. Zhu Y. Chen Q. (2016). Structural basis for the phosphorylation of FUNDC1 LIR as a molecular switch of mitophagy. *Autophagy* 12 2363–2373. 10.1080/15548627.2016.1238552 27653272 PMC5173264

[B85] Kubli D. A. Quinsay M. N. Huang C. Lee Y. Gustafsson A. B. (2008). Bnip3 functions as a mitochondrial sensor of oxidative stress during myocardial ischemia and reperfusion. *Am. J. Physiol. Heart Circ. Physiol.* 295 H2025–H2031. 10.1152/ajpheart.00552.2008 18790835 PMC2614576

[B86] Kumar A. Chaugule V. K. Condos T. E. C. Barber K. R. Johnson C. Toth R. (2017). Parkin-phosphoubiquitin complex reveals cryptic ubiquitin-binding site required for RBR ligase activity. *Nat. Struct. Mol. Biol.* 24 475–483. 10.1038/nsmb.3400 28414322 PMC5420311

[B87] Kumar R. Bukowski M. J. Wider J. M. Reynolds C. A. Calo L. Lepore B. (2016). Mitochondrial dynamics following global cerebral ischemia. *Mol. Cell. Neurosci.* 76 68–75. 10.1016/j.mcn.2016.08.010 27567688 PMC5056829

[B88] Kushnareva Y. E. Gerencser A. A. Bossy B. Ju W. K. White A. D. Waggoner J. (2013). Loss of OPA1 disturbs cellular calcium homeostasis and sensitizes for excitotoxicity. *Cell Death Differ.* 20 353–365. 10.1038/cdd.2012.128 23138851 PMC3554330

[B89] Lagouge M. Mourier A. Lee H. J. Spåhr H. Wai T. Kukat C. (2015). SLIRP regulates the rate of mitochondrial protein synthesis and protects LRPPRC from degradation. *PLoS Genet.* 11:e1005423. 10.1371/journal.pgen.1005423 26247782 PMC4527767

[B90] Lan B. Zhao H. He Y. Zhao Z. Wang N. Gao Y. (2022). Inhibition of human peptide deformylase by actinonin sensitizes glioblastoma cells to temozolomide chemotherapy. *Exp. Cell Res.* 420:113358. 10.1016/j.yexcr.2022.113358 36116558

[B91] Lee J. Y. Nagano Y. Taylor J. P. Lim K. L. Yao T. P. (2010). Disease-causing mutations in parkin impair mitochondrial ubiquitination, aggregation, and HDAC6-dependent mitophagy. *J. Cell Biol.* 189 671–679. 10.1083/jcb.201001039 20457763 PMC2872903

[B92] Lee S. Sterky F. H. Mourier A. Terzioglu M. Cullheim S. Olson L. (2012). Mitofusin 2 is necessary for striatal axonal projections of midbrain dopamine neurons. *Hum. Mol. Genet.* 21 4827–4835. 10.1093/hmg/dds352 22914740

[B93] Lemasters J. J. Zhong Z. (2018). Mitophagy in hepatocytes: Types, initiators and role in adaptive ethanol metabolism ✩. *Liver Res.* 2 125–132. 10.1016/j.livres.2018.09.005 31157120 PMC6541449

[B94] Li Y. J. Cao Y. L. Feng J. X. Qi Y. Meng S. Yang J. F. (2019). Structural insights of human mitofusin-2 into mitochondrial fusion and CMT2A onset. *Nat. Commun.* 10:4914. 10.1038/s41467-019-12912-0 31664033 PMC6820788

[B95] Lin X. H. Qiu B. Q. Ma M. Zhang R. Hsu S. J. Liu H. H. (2020). Suppressing DRP1-mediated mitochondrial fission and mitophagy increases mitochondrial apoptosis of hepatocellular carcinoma cells in the setting of hypoxia. *Oncogenesis* 9:67. 10.1038/s41389-020-00251-5 32661251 PMC7359348

[B96] Liu L. Feng D. Chen G. Chen M. Zheng Q. Song P. (2012). Mitochondrial outer-membrane protein FUNDC1 mediates hypoxia-induced mitophagy in mammalian cells. *Nat. Cell Biol.* 14 177–185. 10.1038/ncb2422 22267086

[B97] Liu Y. Lear T. B. Verma M. Wang K. Z. Otero P. A. McKelvey A. C. (2020). Chemical inhibition of FBXO7 reduces inflammation and confers neuroprotection by stabilizing the mitochondrial kinase PINK1. *JCI Insight* 5:e131834. 10.1172/jci.insight.131834 32493843 PMC7308049

[B98] Losón O. C. Song Z. Chen H. Chan D. C. (2013). Fis1, Mff, MiD49, and MiD51 mediate Drp1 recruitment in mitochondrial fission. *Mol. Biol. Cell* 24 659–667. 10.1091/mbc.E12-10-0721 23283981 PMC3583668

[B99] Lücking C. B. Abbas N. Dürr A. Bonifati V. Bonnet A. M. de Broucker T. (1998). Homozygous deletions in parkin gene in European and North African families with autosomal recessive juvenile parkinsonism. The European consortium on genetic susceptibility in Parkinson’s disease and the French Parkinson’s disease genetics study group. *Lancet* 352 1355–1356. 10.1016/s0140-6736(05)60746-5 9802278

[B100] Lv M. Wang C. Li F. Peng J. Wen B. Gong Q. (2017). Structural insights into the recognition of phosphorylated FUNDC1 by LC3B in mitophagy. *Protein Cell* 8 25–38. 10.1007/s13238-016-0328-8 27757847 PMC5233613

[B101] Mach J. Poliak P. Matusková A. Zárský V. Janata J. Lukes J. (2013). An advanced system of the mitochondrial processing peptidase and core protein family in *Trypanosoma brucei* and multiple origins of the core I subunit in eukaryotes. *Genome Biol. Evol.* 5 860–875. 10.1093/gbe/evt056 23563972 PMC3673636

[B102] Manczak M. Reddy P. H. (2012). Abnormal interaction between the mitochondrial fission protein Drp1 and hyperphosphorylated tau in Alzheimer’s disease neurons: Implications for mitochondrial dysfunction and neuronal damage. *Hum. Mol. Genet.* 21 2538–2547. 10.1093/hmg/dds072 22367970 PMC3349426

[B103] Martin L. J. (2010). Mitochondrial and cell death mechanisms in neurodegenerative diseases. *Pharmaceuticals* 3 839–915. 10.3390/ph3040839 21258649 PMC3023298

[B104] Matsushima Y. Hirofuji Y. Aihara M. Yue S. Uchiumi T. Kaguni L. S. (2017). *Drosophila* protease ClpXP specifically degrades DmLRPPRC1 controlling mitochondrial mRNA and translation. *Sci. Rep.* 7:8315. 10.1038/s41598-017-08088-6 28814717 PMC5559520

[B105] McLelland G. L. Lee S. A. McBride H. M. Fon E. A. (2016). Syntaxin-17 delivers PINK1/parkin-dependent mitochondrial vesicles to the endolysosomal system. *J. Cell Biol.* 214 275–291. 10.1083/jcb.201603105 27458136 PMC4970327

[B106] McLelland G. L. Soubannier V. Chen C. X. McBride H. M. Fon E. A. (2014). Parkin and PINK1 function in a vesicular trafficking pathway regulating mitochondrial quality control. *EMBO J.* 33 282–295. 10.1002/embj.201385902 24446486 PMC3989637

[B107] Meissner C. Lorenz H. Weihofen A. Selkoe D. J. Lemberg M. K. (2011). The mitochondrial intramembrane protease PARL cleaves human Pink1 to regulate Pink1 trafficking. *J. Neurochem.* 117 856–867. 10.1111/j.1471-4159.2011.07253.x 21426348

[B108] Mergenthaler P. Lindauer U. Dienel G. A. Meisel A. (2013). Sugar for the brain: The role of glucose in physiological and pathological brain function. *Trends Neurosci.* 36 587–597. 10.1016/j.tins.2013.07.001 23968694 PMC3900881

[B109] Mishra P. Carelli V. Manfredi G. Chan D. C. (2014). Proteolytic cleavage of Opa1 stimulates mitochondrial inner membrane fusion and couples fusion to oxidative phosphorylation. *Cell Metab.* 19 630–641. 10.1016/j.cmet.2014.03.011 24703695 PMC4018240

[B110] Misko A. L. Sasaki Y. Tuck E. Milbrandt J. Baloh R. H. (2012). Mitofusin2 mutations disrupt axonal mitochondrial positioning and promote axon degeneration. *J. Neurosci.* 32 4145–4155. 10.1523/jneurosci.6338-11.2012 22442078 PMC3319368

[B111] Miyamoto Y. Kitamura N. Nakamura Y. Futamura M. Miyamoto T. Yoshida M. (2011). Possible existence of lysosome-like organella within mitochondria and its role in mitochondrial quality control. *PLoS One* 6:e16054. 10.1371/journal.pone.0016054 21264221 PMC3022026

[B112] Mohammed I. Schmitz K. A. Schenck N. Balasopoulos D. Topitsch A. Maier T. (2022). Catalytic cycling of human mitochondrial Lon protease. *Structure* 30 1254–1268.e7. 10.1016/j.str.2022.06.006 35870450

[B113] Mokranjac D. Neupert W. (2007). Protein import into isolated mitochondria. *Methods Mol. Biol.* 372 277–286. 10.1007/978-1-59745-365-3_20 18314733

[B114] Morgenstern M. Stiller S. B. Lübbert P. Peikert C. D. Dannenmaier S. Drepper F. (2017). Definition of a high-confidence mitochondrial proteome at quantitative scale. *Cell Rep.* 19 2836–2852. 10.1016/j.celrep.2017.06.014 28658629 PMC5494306

[B115] Mossmann D. Meisinger C. Vögtle F. N. (2012). Processing of mitochondrial presequences. *Biochim. Biophys. Acta* 1819 1098–1106. 10.1016/j.bbagrm.2011.11.007 22172993

[B116] Nakamura Y. Kitamura N. Shinogi D. Yoshida M. Goda O. Murai R. (2012). BNIP3 and NIX mediate Mieap-induced accumulation of lysosomal proteins within mitochondria. *PLoS One* 7:e30767. 10.1371/journal.pone.0030767 22292033 PMC3266916

[B117] Nakano N. Ikeda H. O. Hasegawa T. Muraoka Y. Iwai S. Tsuruyama T. (2016). Neuroprotective effects of VCP modulators in mouse models of glaucoma. *Heliyon* 2:e00096. 10.1016/j.heliyon.2016.e00096 27441270 PMC4946081

[B118] Narendra D. P. Jin S. M. Tanaka A. Suen D. F. Gautier C. A. Shen J. (2010). PINK1 is selectively stabilized on impaired mitochondria to activate Parkin. *PLoS Biol.* 8:e1000298. 10.1371/journal.pbio.1000298 20126261 PMC2811155

[B119] Nguyen D. Alavi M. V. Kim K. Y. Kang T. Scott R. T. Noh Y. H. (2011). A new vicious cycle involving glutamate excitotoxicity, oxidative stress and mitochondrial dynamics. *Cell Death Dis.* 2:e240. 10.1038/cddis.2011.117 22158479 PMC3252734

[B120] Novak I. Kirkin V. McEwan D. G. Zhang J. Wild P. Rozenknop A. (2010). Nix is a selective autophagy receptor for mitochondrial clearance. *EMBO Rep.* 11 45–51. 10.1038/embor.2009.256 20010802 PMC2816619

[B121] Oh H. Prevot T. D. Newton D. Sibille E. (2021). From serendipity to rational drug design in brain disorders: In silico, in vitro, and in vivo approaches. *Curr. Opin. Pharmacol.* 60 177–182. 10.1016/j.coph.2021.07.012 34461562

[B122] Okatsu K. Koyano F. Kimura M. Kosako H. Saeki Y. Tanaka K. (2015). Phosphorylated ubiquitin chain is the genuine Parkin receptor. *J. Cell Biol.* 209 111–128. 10.1083/jcb.201410050 25847540 PMC4395490

[B123] Okatsu K. Oka T. Iguchi M. Imamura K. Kosako H. Tani N. (2012). PINK1 autophosphorylation upon membrane potential dissipation is essential for Parkin recruitment to damaged mitochondria. *Nat. Commun.* 3:1016. 10.1038/ncomms2016 22910362 PMC3432468

[B124] Onishi M. Yamano K. Sato M. Matsuda N. Okamoto K. (2021). Molecular mechanisms and physiological functions of mitophagy. *EMBO J.* 40:e104705. 10.15252/embj.2020104705 33438778 PMC7849173

[B125] Ordureau A. Heo J. M. Duda D. M. Paulo J. A. Olszewski J. L. Yanishevski D. (2015). Defining roles of PARKIN and ubiquitin phosphorylation by PINK1 in mitochondrial quality control using a ubiquitin replacement strategy. *Proc. Natl. Acad. Sci. U.S.A.* 112 6637–6642. 10.1073/pnas.1506593112 25969509 PMC4450373

[B126] Osellame L. D. Singh A. P. Stroud D. A. Palmer C. S. Stojanovski D. Ramachandran R. (2016). Cooperative and independent roles of the Drp1 adaptors Mff, MiD49 and MiD51 in mitochondrial fission. *J. Cell Sci.* 129 2170–2181. 10.1242/jcs.185165 27076521 PMC6919635

[B127] Otera H. Miyata N. Kuge O. Mihara K. (2016). Drp1-dependent mitochondrial fission via MiD49/51 is essential for apoptotic cristae remodeling. *J. Cell Biol.* 212 531–544. 10.1083/jcb.201508099 26903540 PMC4772499

[B128] Otera H. Wang C. Cleland M. M. Setoguchi K. Yokota S. Youle R. J. (2010). Mff is an essential factor for mitochondrial recruitment of Drp1 during mitochondrial fission in mammalian cells. *J. Cell Biol.* 191 1141–1158. 10.1083/jcb.201007152 21149567 PMC3002033

[B129] Ott M. Amunts A. Brown A. (2016). Organization and regulation of mitochondrial protein synthesis. *Annu. Rev. Biochem.* 85 77–101. 10.1146/annurev-biochem-060815-014334 26789594

[B130] Park S. W. Kim K. Y. Lindsey J. D. Dai Y. Heo H. Nguyen D. H. (2011). A selective inhibitor of drp1, mdivi-1, increases retinal ganglion cell survival in acute ischemic mouse retina. *Invest. Ophthalmol. Vis. Sci.* 52 2837–2843. 10.1167/iovs.09-5010 21372007 PMC3088566

[B131] Peter B. Waddington C. L. Oláhová M. Sommerville E. W. Hopton S. Pyle A. (2018). Defective mitochondrial protease LonP1 can cause classical mitochondrial disease. *Hum. Mol. Genet.* 27 1743–1753. 10.1093/hmg/ddy080 29518248 PMC5932559

[B132] Pham A. H. Meng S. Chu Q. N. Chan D. C. (2012). Loss of Mfn2 results in progressive, retrograde degeneration of dopaminergic neurons in the nigrostriatal circuit. *Hum. Mol. Genet.* 21 4817–4826. 10.1093/hmg/dds311 22859504 PMC3607482

[B133] Pierson T. M. Adams D. Bonn F. Martinelli P. Cherukuri P. F. Teer J. K. (2011). Whole-exome sequencing identifies homozygous AFG3L2 mutations in a spastic ataxia-neuropathy syndrome linked to mitochondrial m-AAA proteases. *PLoS Genet.* 7:e1002325. 10.1371/journal.pgen.1002325 22022284 PMC3192828

[B134] Poveda-Huertes D. Mulica P. Vögtle F. N. (2017). The versatility of the mitochondrial presequence processing machinery: Cleavage, quality control and turnover. *Cell Tissue Res.* 367 73–81. 10.1007/s00441-016-2492-9 27595151

[B135] Priesnitz C. Becker T. (2018). Pathways to balance mitochondrial translation and protein import. *Genes Dev.* 32 1285–1296. 10.1101/gad.316547.118 30275044 PMC6169841

[B136] Puchades C. Ding B. Song A. Wiseman R. L. Lander G. C. Glynn S. E. (2019). Unique structural features of the mitochondrial AAA+ protease AFG3L2 reveal the molecular basis for activity in health and disease. *Mol. Cell* 75 1073–1085.e6. 10.1016/j.molcel.2019.06.016 31327635 PMC6731152

[B137] Qi Y. Yan L. Yu C. Guo X. Zhou X. Hu X. (2016). Structures of human mitofusin 1 provide insight into mitochondrial tethering. *J. Cell Biol.* 215 621–629. 10.1083/jcb.201609019 27920125 PMC5147005

[B138] Rainbolt T. K. Atanassova N. Genereux J. C. Wiseman R. L. (2013). Stress-regulated translational attenuation adapts mitochondrial protein import through Tim17A degradation. *Cell Metab.* 18 908–919. 10.1016/j.cmet.2013.11.006 24315374 PMC3904643

[B139] Ramelot T. A. Yang Y. Sahu I. D. Lee H. W. Xiao R. Lorigan G. A. (2013). NMR structure and MD simulations of the AAA protease intermembrane space domain indicates peripheral membrane localization within the hexaoligomer. *FEBS Lett.* 587 3522–3528. 10.1016/j.febslet.2013.09.009 24055473 PMC4043124

[B140] Rangaraju V. Calloway N. Ryan T. A. (2014). Activity-driven local ATP synthesis is required for synaptic function. *Cell* 156 825–835. 10.1016/j.cell.2013.12.042 24529383 PMC3955179

[B141] Rappold P. M. Cui M. Grima J. C. Fan R. Z. de Mesy-Bentley K. L. Chen L. (2014). Drp1 inhibition attenuates neurotoxicity and dopamine release deficits in vivo. *Nat. Commun.* 5:5244. 10.1038/ncomms6244 25370169 PMC4223875

[B142] Ray R. Chen G. Vande Velde C. Cizeau J. Park J. H. Reed J. C. (2000). BNIP3 heterodimerizes with Bcl-2/Bcl-X(L) and induces cell death independent of a Bcl-2 homology 3 (BH3) domain at both mitochondrial and nonmitochondrial sites. *J. Biol. Chem.* 275 1439–1448. 10.1074/jbc.275.2.1439 10625696

[B143] Reddy P. H. Manczak M. Yin X. (2017). Mitochondria-division inhibitor 1 protects against amyloid-β induced mitochondrial fragmentation and synaptic damage in Alzheimer’s disease. *J. Alzheimers Dis.* 58 147–162. 10.3233/jad-170051 28409745 PMC5444307

[B144] Regen F. Hellmann-Regen J. Costantini E. Reale M. (2017). Neuroinflammation and Alzheimer’s disease: Implications for microglial activation. *Curr. Alzheimer Res.* 14 1140–1148. 28164764 10.2174/1567205014666170203141717

[B145] Richter V. Palmer C. S. Osellame L. D. Singh A. P. Elgass K. Stroud D. A. (2014). Structural and functional analysis of MiD51, a dynamin receptor required for mitochondrial fission. *J. Cell Biol.* 204 477–486. 10.1083/jcb.201311014 24515348 PMC3926961

[B146] Rikka S. Quinsay M. N. Thomas R. L. Kubli D. A. Zhang X. Murphy A. N. (2011). Bnip3 impairs mitochondrial bioenergetics and stimulates mitochondrial turnover. *Cell Death Differ.* 18 721–731. 10.1038/cdd.2010.146 21278801 PMC3058880

[B147] Roca-Agujetas V. Barbero-Camps E. de Dios C. Podlesniy P. Abadin X. Morales A. (2021). Cholesterol alters mitophagy by impairing optineurin recruitment and lysosomal clearance in Alzheimer’s disease. *Mol. Neurodegener.* 16:15. 10.1186/s13024-021-00435-6 33685483 PMC7941983

[B148] Rogov V. V. Suzuki H. Marinković M. Lang V. Kato R. Kawasaki M. (2017). Phosphorylation of the mitochondrial autophagy receptor Nix enhances its interaction with LC3 proteins. *Sci. Rep.* 7:1131. 10.1038/s41598-017-01258-6 28442745 PMC5430633

[B149] Rojo M. Legros F. Chateau D. Lombès A. (2002). Membrane topology and mitochondrial targeting of mitofusins, ubiquitous mammalian homologs of the transmembrane GTPase Fzo. *J. Cell Sci.* 115 1663–1674. 10.1242/jcs.115.8.1663 11950885

[B150] Saita S. Tatsuta T. Lampe P. A. König T. Ohba Y. Langer T. (2018). PARL partitions the lipid transfer protein STARD7 between the cytosol and mitochondria. *EMBO J.* 37:e97909. 10.15252/embj.201797909 29301859 PMC5813258

[B151] Sandoval H. Thiagarajan P. Dasgupta S. K. Schumacher A. Prchal J. T. Chen M. (2008). Essential role for Nix in autophagic maturation of erythroid cells. *Nature* 454 232–235. 10.1038/nature07006 18454133 PMC2570948

[B152] Santel A. Fuller M. T. (2001). Control of mitochondrial morphology by a human mitofusin. *J. Cell Sci.* 114 867–874. 10.1242/jcs.114.5.867 11181170

[B153] Seo J. H. Rivadeneira D. B. Caino M. C. Chae Y. C. Speicher D. W. Tang H. Y. (2016). The mitochondrial unfoldase-peptidase complex ClpXP controls bioenergetics stress and metastasis. *PLoS Biol.* 14:e1002507. 10.1371/journal.pbio.1002507 27389535 PMC4936714

[B154] Sepuri N. B. V. Angireddy R. Srinivasan S. Guha M. Spear J. Lu B. (2017). Mitochondrial LON protease-dependent degradation of cytochrome c oxidase subunits under hypoxia and myocardial ischemia. *Biochim. Biophys. Acta Bioenerget.* 1858 519–528. 10.1016/j.bbabio.2017.04.003 28442264 PMC5507603

[B155] Shamseldin H. E. Alshammari M. Al-Sheddi T. Salih M. A. Alkhalidi H. Kentab A. (2012). Genomic analysis of mitochondrial diseases in a consanguineous population reveals novel candidate disease genes. *J. Med. Genet.* 49 234–241. 10.1136/jmedgenet-2012-100836 22499341

[B156] Shiba-Fukushima K. Imai Y. Yoshida S. Ishihama Y. Kanao T. Sato S. (2012). PINK1-mediated phosphorylation of the Parkin ubiquitin-like domain primes mitochondrial translocation of Parkin and regulates mitophagy. *Sci. Rep.* 2:1002. 10.1038/srep01002 23256036 PMC3525937

[B157] Simon D. Laloo B. Barillot M. Barnetche T. Blanchard C. Rooryck C. (2010). A mutation in the 3’-UTR of the HDAC6 gene abolishing the post-transcriptional regulation mediated by hsa-miR-433 is linked to a new form of dominant X-linked chondrodysplasia. *Hum. Mol. Genet.* 19 2015–2027. 10.1093/hmg/ddq083 20181727

[B158] Song Z. Ghochani M. McCaffery J. M. Frey T. G. Chan D. C. (2009). Mitofusins and OPA1 mediate sequential steps in mitochondrial membrane fusion. *Mol. Biol. Cell* 20 3525–3532. 10.1091/mbc.e09-03-0252 19477917 PMC2719570

[B159] Soubannier V. McLelland G. L. Zunino R. Braschi E. Rippstein P. Fon E. A. (2012). A vesicular transport pathway shuttles cargo from mitochondria to lysosomes. *Curr. Biol.* 22 135–141. 10.1016/j.cub.2011.11.057 22226745

[B160] Sousa J. S. D’Imprima E. Vonck J. (2018). Mitochondrial respiratory chain complexes. *Subcell. Biochem.* 87 167–227. 10.1007/978-981-10-7757-9_7 29464561

[B161] Sprenger H. G. Wani G. Hesseling A. König T. Patron M. MacVicar T. (2019). Loss of the mitochondrial i-AAA protease YME1L leads to ocular dysfunction and spinal axonopathy. *EMBO Mol. Med.* 11:e9288. 10.15252/emmm.201809288 30389680 PMC6328943

[B162] Stefanatos R. Sanz A. (2018). The role of mitochondrial ROS in the aging brain. *FEBS Lett.* 592 743–758. 10.1002/1873-3468.12902 29106705

[B163] Stojanovski D. Koutsopoulos O. S. Okamoto K. Ryan M. T. (2004). Levels of human Fis1 at the mitochondrial outer membrane regulate mitochondrial morphology. *J. Cell Sci.* 117 1201–1210. 10.1242/jcs.01058 14996942

[B164] Strauss K. A. Jinks R. N. Puffenberger E. G. Venkatesh S. Singh K. Cheng I. (2015). CODAS syndrome is associated with mutations of LONP1, encoding mitochondrial AAA+ Lon protease. *Am. J. Hum. Genet.* 96 121–135. 10.1016/j.ajhg.2014.12.003 25574826 PMC4289676

[B165] Sultana R. Mecocci P. Mangialasche F. Cecchetti R. Baglioni M. Butterfield D. A. (2011). Increased protein and lipid oxidative damage in mitochondria isolated from lymphocytes from patients with Alzheimer’s disease: Insights into the role of oxidative stress in Alzheimer’s disease and initial investigations into a potential biomarker for this dementing disorder. *J. Alzheimers Dis.* 24 77–84. 10.3233/jad-2011-101425 21383494

[B166] Suzuki M. Jeong S. Y. Karbowski M. Youle R. J. Tjandra N. (2003). The solution structure of human mitochondria fission protein Fis1 reveals a novel TPR-like helix bundle. *J. Mol. Biol.* 334 445–458. 10.1016/j.jmb.2003.09.064 14623186

[B167] Tremel S. Ohashi Y. Morado D. R. Bertram J. Perisic O. Brandt L. T. L. (2021). Structural basis for VPS34 kinase activation by Rab1 and Rab5 on membranes. *Nat. Commun.* 12:1564. 10.1038/s41467-021-21695-2 33692360 PMC7946940

[B168] Tsuboyama K. Koyama-Honda I. Sakamaki Y. Koike M. Morishita H. Mizushima N. (2016). The ATG conjugation systems are important for degradation of the inner autophagosomal membrane. *Science* 354 1036–1041. 10.1126/science.aaf6136 27885029

[B169] Valente E. M. Abou-Sleiman P. M. Caputo V. Muqit M. M. Harvey K. Gispert S. (2004). Hereditary early-onset Parkinson’s disease caused by mutations in PINK1. *Science* 304 1158–1160. 10.1126/science.1096284 15087508

[B170] Valente E. M. Bentivoglio A. R. Dixon P. H. Ferraris A. Ialongo T. Frontali M. (2001). Localization of a novel locus for autosomal recessive early-onset parkinsonism, PARK6, on human chromosome 1p35-p36. *Am. J. Hum. Genet.* 68 895–900. 10.1086/319522 11254447 PMC1275643

[B171] Valente E. M. Brancati F. Ferraris A. Graham E. A. Davis M. B. Breteler M. M. (2002). PARK6-linked parkinsonism occurs in several European families. *Ann. Neurol.* 51 14–18. 11782979

[B172] VanItallie T. B. (2019). Traumatic brain injury (TBI) in collision sports: Possible mechanisms of transformation into chronic traumatic encephalopathy (CTE). *Metabolism* 100S:153943. 10.1016/j.metabol.2019.07.007 31610856

[B173] Vanstone J. R. Smith A. M. McBride S. Naas T. Holcik M. Antoun G. (2016). DNM1L-related mitochondrial fission defect presenting as refractory epilepsy. *Eur. J. Hum. Genet.* 24 1084–1088. 10.1038/ejhg.2015.243 26604000 PMC5070894

[B174] Vercellino I. Sazanov L. A. (2022). The assembly, regulation and function of the mitochondrial respiratory chain. *Nat. Rev. Mol. Cell Biol.* 23 141–161. 10.1038/s41580-021-00415-0 34621061

[B175] Verrigni D. Di Nottia M. Ardissone A. Baruffini E. Nasca A. Legati A. (2019). Clinical-genetic features and peculiar muscle histopathology in infantile DNM1L-related mitochondrial epileptic encephalopathy. *Hum. Mutat.* 40 601–618. 10.1002/humu.23729 30801875

[B176] Wai T. García-Prieto J. Baker M. J. Merkwirth C. Benit P. Rustin P. (2015). Imbalanced OPA1 processing and mitochondrial fragmentation cause heart failure in mice. *Science* 350:aad0116. 10.1126/science.aad0116 26785494

[B177] Wang J. Hansen K. Edwards R. Van Houten B. Qian W. (2015). Mitochondrial division inhibitor 1 (mdivi-1) enhances death receptor-mediated apoptosis in human ovarian cancer cells. *Biochem. Biophys. Res. Commun.* 456 7–12. 10.1016/j.bbrc.2014.11.010 25446129 PMC4297922

[B178] Wang L. Klionsky D. J. Shen H. M. (2022). The emerging mechanisms and functions of microautophagy. *Nat. Rev. Mol. Cell Biol.* [Epub ahead of print]. 10.1038/s41580-022-00529-z 36097284

[B179] Wang W. Yin J. Ma X. Zhao F. Siedlak S. L. Wang Z. (2017). Inhibition of mitochondrial fragmentation protects against Alzheimer’s disease in rodent model. *Hum. Mol. Genet.* 26 4118–4131. 10.1093/hmg/ddx299 28973308 PMC5886251

[B180] Wang X. Su B. Lee H. G. Li X. Perry G. Smith M. A. (2009). Impaired balance of mitochondrial fission and fusion in Alzheimer’s disease. *J. Neurosci.* 29 9090–9103. 10.1523/jneurosci.1357-09.2009 19605646 PMC2735241

[B181] Wang Y. Zhan G. Cai Z. Jiao B. Zhao Y. Li S. (2021). Vagus nerve stimulation in brain diseases: Therapeutic applications and biological mechanisms. *Neurosci. Biobehav. Rev.* 127 37–53. 10.1016/j.neubiorev.2021.04.018 33894241

[B182] Waterham H. R. Koster J. van Roermund C. W. Mooyer P. A. Wanders R. J. Leonard J. V. (2007). A lethal defect of mitochondrial and peroxisomal fission. *N. Engl. J. Med.* 356 1736–1741. 10.1056/NEJMoa064436 17460227

[B183] Weckbecker D. Longen S. Riemer J. Herrmann J. M. (2012). Atp23 biogenesis reveals a chaperone-like folding activity of Mia40 in the IMS of mitochondria. *EMBO J.* 31 4348–4358. 10.1038/emboj.2012.263 22990235 PMC3501227

[B184] Williams P. A. Morgan J. E. Votruba M. (2011). Mouse models of dominant optic atrophy: What do they tell us about the pathophysiology of visual loss? *Vision Res.* 51 229–234. 10.1016/j.visres.2010.08.031 20801145

[B185] Wu W. Tian W. Hu Z. Chen G. Huang L. Li W. (2014). ULK1 translocates to mitochondria and phosphorylates FUNDC1 to regulate mitophagy. *EMBO Rep.* 15 566–575. 10.1002/embr.201438501 24671035 PMC4210082

[B186] Xiao X. Hu Y. Quirós P. M. Wei Q. López-Otín C. Dong Z. (2014). OMA1 mediates OPA1 proteolysis and mitochondrial fragmentation in experimental models of ischemic kidney injury. *Am. J. Physiol. Renal Physiol.* 306 F1318–F1326. 10.1152/ajprenal.00036.2014 24671334 PMC4042105

[B187] Xie J. Shen Z. Anraku Y. Kataoka K. Chen X. (2019). Nanomaterial-based blood-brain-barrier (BBB) crossing strategies. *Biomaterials* 224:119491. 10.1016/j.biomaterials.2019.119491 31546096 PMC6915305

[B188] Xu S. Peng G. Wang Y. Fang S. Karbowski M. (2011). The AAA-ATPase p97 is essential for outer mitochondrial membrane protein turnover. *Mol. Biol. Cell* 22 291–300. 10.1091/mbc.E10-09-0748 21118995 PMC3031461

[B189] Yan L. Qi Y. Huang X. Yu C. Lan L. Guo X. (2018). Structural basis for GTP hydrolysis and conformational change of MFN1 in mediating membrane fusion. *Nat. Struct. Mol. Biol.* 25 233–243. 10.1038/s41594-018-0034-8 29483649

[B190] Yang D. Zhou Q. Labroska V. Qin S. Darbalaei S. Wu Y. (2021). G protein-coupled receptors: Structure- and function-based drug discovery. *Signal Transduct. Target. Ther.* 6:7. 10.1038/s41392-020-00435-w 33414387 PMC7790836

[B191] Ye X. Sun X. Starovoytov V. Cai Q. (2015). Parkin-mediated mitophagy in mutant hAPP neurons and Alzheimer’s disease patient brains. *Hum. Mol. Genet.* 24 2938–2951. 10.1093/hmg/ddv056 25678552 PMC4406302

[B192] Yin F. Sancheti H. Patil I. Cadenas E. (2016). Energy metabolism and inflammation in brain aging and Alzheimer’s disease. *Free Radic. Biol. Med.* 100 108–122. 10.1016/j.freeradbiomed.2016.04.200 27154981 PMC5094909

[B193] Yoon Y. Krueger E. W. Oswald B. J. McNiven M. A. (2003). The mitochondrial protein hFis1 regulates mitochondrial fission in mammalian cells through an interaction with the dynamin-like protein DLP1. *Mol. Cell. Biol.* 23 5409–5420. 10.1128/mcb.23.15.5409-5420.2003 12861026 PMC165727

[B194] Yu C. Zhao J. Yan L. Qi Y. Guo X. Lou Z. (2020). Structural insights into G domain dimerization and pathogenic mutation of OPA1. *J. Cell Biol.* 219:e201907098. 10.1083/jcb.201907098 32379273 PMC7337494

[B195] Yue Z. Y. Dong H. Wang Y. F. Liu Y. Song C. Y. Yang W. C. (2015). Propofol prevents neuronal mtDNA deletion and cerebral damage due to ischemia/reperfusion injury in rats. *Brain Res.* 1594 108–114. 10.1016/j.brainres.2014.10.016 25451088

[B196] Zhang C. Shang G. Gui X. Zhang X. Bai X. C. Chen Z. J. (2019). Structural basis of STING binding with and phosphorylation by TBK1. *Nature* 567 394–398. 10.1038/s41586-019-1000-2 30842653 PMC6862768

[B197] Zhao H. Luo Y. Chen L. Zhang Z. Shen C. Li Y. (2018a). Sirt3 inhibits cerebral ischemia-reperfusion injury through normalizing Wnt/β-catenin pathway and blocking mitochondrial fission. *Cell Stress Chaperones* 23 1079–1092. 10.1007/s12192-018-0917-y 29862442 PMC6111081

[B198] Zhao H. Pan W. Chen L. Luo Y. Xu R. (2018b). Nur77 promotes cerebral ischemia-reperfusion injury via activating INF2-mediated mitochondrial fragmentation. *J. Mol. Histol.* 49 599–613. 10.1007/s10735-018-9798-8 30298449

[B199] Zhao Y. Zhang J. Zheng Y. Zhang Y. Zhang X. J. Wang H. (2021). NAD(+) improves cognitive function and reduces neuroinflammation by ameliorating mitochondrial damage and decreasing ROS production in chronic cerebral hypoperfusion models through Sirt1/PGC-1α pathway. *J. Neuroinflammation* 18:207. 10.1186/s12974-021-02250-8 34530866 PMC8444613

[B200] Zhu H. (2020). Big data and artificial intelligence modeling for drug discovery. *Annu. Rev. Pharmacol. Toxicol.* 60 573–589. 10.1146/annurev-pharmtox-010919-023324 31518513 PMC7010403

[B201] Zhu Y. Massen S. Terenzio M. Lang V. Chen-Lindner S. Eils R. (2013). Modulation of serines 17 and 24 in the LC3-interacting region of Bnip3 determines pro-survival mitophagy versus apoptosis. *J. Biol. Chem.* 288 1099–1113. 10.1074/jbc.M112.399345 23209295 PMC3542995

[B202] Zorov D. B. Juhaszova M. Sollott S. J. (2014). Mitochondrial reactive oxygen species (ROS) and ROS-induced ROS release. *Physiol. Rev.* 94 909–950. 10.1152/physrev.00026.2013 24987008 PMC4101632

[B203] Zurita Rendón O. Shoubridge E. A. (2018). LONP1 is required for maturation of a subset of mitochondrial proteins, and its loss elicits an integrated stress response. *Mol. Cell. Biol.* 38:e00412-17. 10.1128/mcb.00412-17 30061372 PMC6168981

